# On the Use of Deuterated Organic Solvents without TMS to Report ^1^H/^13^C NMR Spectral Data of Organic Compounds: Current State of the Method, Its Pitfalls and Benefits, and Related Issues

**DOI:** 10.3390/molecules28114369

**Published:** 2023-05-26

**Authors:** Ryszard B. Nazarski

**Affiliations:** Theoretical and Structural Chemistry Group, Department of Physical Chemistry, Faculty of Chemistry, University of Lodz, 163/165 Pomorska, 90-236 Łódź, Poland; ryszard.nazarski@chemia.uni.lodz.pl

**Keywords:** NMR spectroscopy, tetramethylsilane, internal reference standards, deuterated solvent signals, residual ^1^H signals, the IUPAC unified *Ξ* scale of chemical shifts, MestReNova, GIAO-DFT-NMR

## Abstract

The quite popular, simple but imperfect method of referencing NMR spectra to residual ^1^H and ^13^C signals of TMS-free deuterated organic solvents (referred to as Method A) is critically discussed for six commonly used NMR solvents with respect to their δ_H_ and δ_C_ values that exist in the literature. Taking into account the most reliable data, it was possible to recommend ‘best’ δ_X_ values for such secondary internal standards. The position of these reference points on the δ scale strongly depends on the concentration and type of analyte under study and the solvent medium used. For some solvents, chemically induced shifts (CISs) of residual ^1^H lines were considered, also taking into account the formation of 1:1 molecular complexes (for CDCl_3_). Typical potential errors that can occur as a result of improper application of Method A are considered in detail. An overview of all found δ_X_ values adopted by users of this method revealed a discrepancy of up to 1.9 ppm in δ_C_ reported for CDCl_3_, most likely caused by the CIS mentioned above. The drawbacks of Method A are discussed in relation to the classical use of an internal standard (Method B), two ‘instrumental’ schemes in which Method A is often implicitly applied, that is, the default Method C using ^2^H lock frequencies and Method D based on *Ξ* values, recommended by the IUPAC but only occasionally used for ^1^H/^13^C spectra, and external referencing (Method E). Analysis of current needs and opportunities for NMR spectrometers led to the conclusion that, for the most accurate application of Method A, it is necessary to (a) use dilute solutions in a single NMR solvent and (b) to report δ_X_ data applied for the reference ^1^H/^13^C signals to the nearest 0.001/0.01 ppm to ensure the precise characterization of new synthesized or isolated organic systems, especially those with complex or unexpected structures. However, the use of TMS in Method B is strongly recommended in all such cases.

## 1. Introduction

Nuclear magnetic resonance (NMR) spectroscopy is undoubtedly the most reliable and rich source of information on the structure, dynamics, and reactivity of any chemical system, especially in solution. The use of deuterated solvents to provide the ^2^H field-frequency lock for today’s spectrometers is evident. CDCl_3_ and DMSO-*d*_6_ are the most commonly used due to their very good solubilizing properties. The latter is more versatile but more expensive and difficult to remove from the NMR sample.

The only question that remains is how to relate the resonance signals in the NMR spectra of the analytes under study to proper zero-frequency reference materials, which are critical in this analytical technique. The debate on standardizing NMR chemical shifts is ongoing [1,2]. Interestingly, new reference materials are constantly being proposed, such as relatively chemically inert cubane, which appears to be an ideal internal standard for reactions monitored with ^1^H and/or ^13^C spectra [3]; new related results on this topic and their discussion are presented in Supplementary Materials.

A recently published article by Guzman and Hoye [4] discussed typical situations that chemists face daily during the NMR spectral characterization of new products or isolates from natural resources. Highly reliable δ_H_ data were obtained with a coaxial tube arrangement that shows that internal tetramethylsilane (TMS) is superior to the residual CHCl_3_ signal for routine reference of ^1^H spectra taken in CDCl_3_. It was found that, as a result of intermolecular interactions between TMS (or residual CHCl_3_) and analytes of various types, the lines of the former shift to a higher or lower frequency (lower or higher field) as the concentration of the NMR sample increases, confirming previous results by Hatada and Kitayama [5]. However, this chemically induced shift (CIS) was much smaller for TMS for the vast majority of analytes tested. Other authors [6,7] have already noted work [4] and recognized it as a call to return to the roots of NMR [6], namely to the originally [8] proposed use of TMS as an internal reference standard confirmed in later papers [9,10].

Guzman and Hoye [4] provided beneficially strong evidence for the superiority of using internal TMS for ^1^H NMR spectra taken in CDCl_3_. However, the author felt that a broader look at this convenient but essentially unregulated practice of recording spectra in TMS-free NMR solvents and then reporting the resulting spectral data on the TMS scale is needed. Guzman and Hoye [4] did not relate their study to important ^13^C nuclei and limited themselves to a single solvent (CDCl_3_).

Accordingly, this short review addresses some of the issues that have emerged from a thorough literature review on the subject; and may be of interest to the chemistry community at large. Here, a great set of different ‘reference’ δ_X_ data (X = H and C), applied in the aforementioned referencing practice, is critically discussed for the six most commonly used deuterated solvents [11]. At the same time, other important questions related to this topic are considered. The idea behind this paper was to organize (and in some cases even correct) existing information in the literature on the title method of standardizing NMR spectra and several other closely related issues. To the best of the author’s knowledge, no such coverage of all these topics has been presented to date.

In a recent paper by Napolitano et al. [7], which deals with the chemical shift referencing strategy proposed by them for neat organic liquids using benchtop NMR systems, the following statement was made: “Unfortunately, relinquishing the use of deuterated solvents and other additives such as TMS can give rise to inconsistencies in chemical shift referencing.” The authors wrote this sentence quoting two research papers [4,12] but no review articles. The purpose of this publication is to partially fill the research gap regarding non-aqueous solutions in six selected deuterated solvents.

However, it should be noted that some issues related to the topics discussed here, such as a more accurate analysis of the sources of CISs resulting from changes in the analyte type versus changes involving the reference material used or comparison of results or errors arising from the application of all four Methods A–E (see Table 1), are beyond the scope of this review, which is, in the vast majority, based only on the analysis of data and results already published in the literature.

## 2. Search Results and Discussion

Internal TMS in CDCl_3_ is a perfect standard for δ values of three key NMR nuclei, namely, ^1^H, ^13^C, and ^29^Si. Its strong and sharp line is distinct from other signals in the vast majority of samples analyzed. Therefore, the IUPAC 2001 definition gives δ_X_ = 0 ppm for the ^1^H line of TMS [in a 1% (*v*/*v*) CDCl_3_ solution] as the primary reference for all these nuclei using the common cylindrical NMR tubes [1]. It is worth noting that δ_C_ of 0.74 ppm was found for such a solution using a spherical ampule; hence, this value is free of the effects of bulk magnetic susceptibility (BMS) [13].

However, the use of TMS as a reference material for the spectral characterization of silyl derivatives can sometimes be difficult, especially with older NMR spectrometers, due to the possible overlap of ^1^H resonance signals. A good example of this is the case of compounds containing the *tert*-butyldimethylsilyl (*t*-BuCH_2_Si, TBS) protecting group [14,15,16]. In the first work [14], no method was given to characterize the 400/100 MHz ^1^H/^13^C spectra of the obtained product, while, in the second [15], the corresponding NMR spectra were referenced to residual ^1^H and ^13^C solvent signals used as internal standards (hereafter referred to as two variants of Method A). In contrast, in a recent study [16] conducted on a 600 MHz system, a classical approach with internal TMS (Method B) was applied.

Generally, TMS is fairly neutral to typical organic compounds. However, there may be slight differences in its δ_H_ values, especially in the presence of aromatic systems in the analyzed solutions [5,8,9,10,17,18,19]. Much larger changes were found for its δ_C_ data [19,20,21,22].

### 2.1. Some History

It has been known for about 55 years [23,24,25,26,27,28] that the experimental δ_X_ values are sometimes indirectly calibrated to the TMS line at 0 ppm in NMR solvents using the aforementioned Method A, in which the δ_X_ data were adopted from the available literature [24,25,29,30]. The requirement introduced in most reputable journals to include copies of NMR spectra taken for all new compounds/isolates has revealed the widespread use of this simple but imperfect method.

According to this search, the first compilation of δ_H_ data for residual impurities of available deuterated NMR solvents was performed at Merck Sharp & Dohme of Canada, Ltd. (hereafter referred to as MSD), Pointe-Claire, Dorval, Quebec, Canada. Most likely, it originated as a specification of the supplied solvents to identify the ^1^H lines of their incompletely deuterated components. This data set was already included in a textbook by Silverstein et al., in its 1967 edition [31]. In fact, this year a study [23] was published using the residual ^1^H signal of CDCl_3_ as an internal standard. The extension of the above set to cover the ^13^C data was also carried out by the Isotope Division of MSD using 100/25 MHz ^1^H/^13^C spectra of solutions containing 5% TMS (*v*/*v*). Indeed, two articles [24,32] have been found in which NMR spectra were referenced to solvent signals whose δ_X_s were taken from undated reference data [33] provided by MSD. All these values have also been quoted in some books [26,28,34,35]. Such δ_X_ data were therefore widely available in the second half of the 1980s. For their original list shown in ref. [34], see Table S1 of Supplementary Materials. This data set is still available online (but its source is not provided) [33,36]. Its update [37,38], based on 200/50 MHz NMR spectra taken for more dilute solutions of TMS, has already been carried out at Cambridge Isotope Laboratories, Inc. (Tewksbury, MA, USA), which acquired the assets of MSD Isotopes (its main global competitor) in 1993 from Merck Frosst of Canada. It should be noted that the book [39] was erroneously cited in ref. [38] as the source of reference δ_X_ data instead of ref. [37]. Only the melting and boiling points of the deuterated NMR solvents are given in ref. [37] were taken from this book.

Other similar tables covering TMS-doped solvents have been published in a few books or booklets [5,40,41,42,43,44,45,46,47,48], sometimes with erroneous δ_X_s [47] (see Tables S2 and S4). However, a significant spate of articles reporting NMR data found by Method A did not occur until after the publication of a highly cited work by Gottlieb et al. [49]. It should be highlighted that δ_C_ data for some solvents have been originally published in this work with quite large uncertainties, for example of ±0.06 ppm for CDCl_3_ and DMSO-*d*_6_. Their article, which was in part an update of Fletton and Page’s early paper on ^1^H NMR data only [50], was later expanded to include additional impurities and solvents [51,52]. Similar δ_X_ sets have also been reported by other authors [53,54]. Evidently, in light of all of the above facts, some researchers have found the application of Method A for referencing NMR spectra to be completely correct [5,26,28,55].

Undoubtedly, due to its ease of use, some Ph.D. students around the world were eager to use Method A to record and report NMR spectra. As a result, the determined spectral data found their way into their theses and later into related original research articles. It follows that the reviewers involved were not opposed to this reference scheme. Over time, this became widespread and fully accepted [38,55,56,57,58,59]. Not surprisingly, the past decade has seen a plethora of articles in which chemical shifts were indirectly referenced to TMS, using Method A. Its implicit use in the increasingly applied post-processing NMR spectral data MestReNova program [60] has further popularized this very simple procedure.

### 2.2. Methods for ^1^H/^13^C Chemical Shift Reference

In Hoffman’s article [61], the following can be read: “The common practice today is to measure the chemical shift relative to the solvent peak in the proton spectrum, to the signal of TMS… or to rely on the spectrometer to set the frequency relative to the deuterium signal of the solvent” (referred to herein as Method A, the ^1^H variant of Method B and Method C, respectively). Two additional reference schemes, namely Methods D and E, are also in normal use (see Table 1).

In fact, all current NMR instruments can ‘lock’ on the ^2^H signal of deuterated solvents [5,55,61,62,63,64,65,66,67]; therefore, the addition of internal reference standards is really not required [55,65,66,67]. Such a *by solvent* or *solvent-based scheme* can be used in Method C for all NMR-active nuclei. This approach, which is usually treated as default in the protocols of all modern spectrometers, uses the ^2^H lock frequencies of the NMR solvents (see Table S5) to reference δ_X_ data instead of the ^1^H frequency of TMS (as recommended by the IUPAC). However, as is the case with the others, this approach has some benefits and drawbacks [12,61]. The resulting spectra are typically calibrated using Method A or some third-party post-processing packages, such as the aforementioned MestReNova program [60], which also implicitly uses this procedure, as well as the δ_X_ values of refs. [37,49]. Similar to the case of ref. [38] (*vide supra*), in MestReNova the book [39] is erroneously cited as the source of the reference δ_X_ data instead of ref. [37]. It should also be emphasized that the CIL’s NMR Solvent Data Chart [37] contains such values for pure TMS-doped solvents, which is a completely different case from those that occur in real situations (*vide infra*). If TMS is present in the sample, Method B can be used.

The specific variant of Method C discussed above is the *substitution method*. Its application involves using a separate standard NMR tube with the reference standard and then directly recording the spectrum of the analyte in a solvent without reference material. Any changes in locking and shimming between the two samples should be avoided. However, the results obtained in this way are not accurate, even if the same deuterated solvent is used in both cases, mainly due to the lack of adjustment performed for the second sample and the CIS and/or BMS effects that can also occur for more concentrated solutions [2]. Consequently, the use of this approach is currently not recommended in most situations [68].

The second ‘instrumental’ scheme (Method D), which is very rarely used for ^1^H/^13^C nuclei, is recommended by the IUPAC for indirect referencing of δ_X_s of all NMR-active nuclei. This accurate unified scale of chemical shifts is based on predetermined ratios of appropriate absolute frequencies (*Ξ* values), with a primary reference of TMS in CDCl_3_ (*vide supra*) [1,2,69,70,71,72,73,74,75,76]. However, sometimes, non-TMS signals are used as secondary internal standards [71,72,73]. For example, ^1^H NMR spectra were calibrated to the TMS scale using the central signal of the CHClF_2_ triplet as an internal secondary reference standard set to 7.21 ppm [70]. In turn, in ref. [72], the ^14^N chemical shifts were referenced indirectly using a ^1^H NMR frequency of solid adamantane at 1.8 ppm.

The application of Method D includes several NMR-active nuclei essential in medical and biological sciences, namely ^11^B, ^15^N, ^19^F, and ^31^P [12,69,70,71,72,73,74,75,76]. More specifically, in Bruker NMR systems, having two spectra, for example, ^1^H and, e.g., ^14^N or ^19^F, after referencing the first (in Method A or, much better, using Method B), the second spectrum is usually standardized indirectly [73] with respect to the ^1^H frequency using the *xiref* referencing macro implemented in TopSpin software. Importantly, it avoids the widespread, but now not-recommended, use of primary external standards, namely 15% BF_3_·OEt_2_ in CDCl_3_, neat CH_3_NO_2_, neat CFCl_3_, and 85% H_3_PO_4_, respectively [12,77,78,79], which is usually less accurate and sometimes very dangerous. In fact, it can be assumed that the death of an academic chemist as a result of exposure to liquid Me_2_Hg (external reference for ^199^Hg NMR spectroscopy) accelerated the introduction of this universal procedure at the expense of using the highly problematic Method E [80,81,82]. The application of Method D, which is available in MestReNova [60], is explained in numerous articles [1,2,12,19,61,69,70,71,72,73], books [38,65,83], manuals [60], and websites [74,75,76]. One of the δ_C_ data sets discussed in the Supplementary Materials was found in this way (see footnote *f* to Table S4).

The use of the external references mentioned above is the domain of Method E. In this approach, the reference material (pure liquid or solution) is usually placed in an inner capillary of a two-tube coaxial arrangement. Sometimes, dedicated reference standards have been proposed or used. For example, Batley and Redmond [78] suggested the application of an aqueous solution of tetrahydroxyphosphonium perchlorate as an external secondary ^31^P standard, with δ_P_ = 0.09 ppm, instead of a well-known primary standard, which is 85% phosphoric acid, with δ_P_ = −0.73 ppm, for measurements in aqueous solutions using standard cylindrical NMR tubes. In estimating the two aforementioned reference δ_P_ data, these authors used corrections for the difference in BMS values between the aqueous sample and the reference material. In turn, in the work [84], the ^19^F NMR chemical shifts were referenced against CFCl_3_ using BF_3_∙Et_2_O as an external secondary standard with δ_F_ = −153.0 ppm.

Generally, Method E is currently rather discouraged, as a tedious (usually not very accurate) BMS correction must be used when applied correctly (*vide supra/infra*). Its use is common in different host–guest [85,86] or pH-dependent ^1^H/^13^C NMR titrations [87,88]. However, in this way, somewhat inaccurate δ_X_ data are usually obtained, even when the analyte and reference standard used are dissolved in the same solvent, due to gradual changes in the magnetic susceptibility of the titrated solution when successive aliquots of titrant are added. In general, to nullify the difference in BMS between the two solutions in question, a coaxial arrangement of two cells would have to be applied, with the sample and reference material being placed in two perfectly spherical containers [21,22,77].

The non-typical *use of the magic-angle spinning (MAS) technique* for NMR referencing in solution should still be discussed here for the sake of completeness. The signal positions found by this approach using standard cylindrical tubes are practically free of the effects of BMS and solvent influence. Therefore, with the use of this non-standard method, it was possible to determine the ‘absolute’ δ_X_ values for benzene (C_6_H_6_) or chloroform (CHCl_3_) and TMS (δ_X_ = 0 ppm), as well as their subsequent changes when mixed within these two pairs of pure liquids [89]. The δ_X_ data for TMS in the solutions thus formed were not zero, as expected. The following data, δ_H_ = 0.100 ppm and δ_C_ = 0.554 ppm, were found for the CHCl_3_ solution.

Indeed, the chemical literature is highly non-heterogeneous with regard to NMR referencing, as noted by Pauli et al. [57]. Various aspects of this topic are described on the Chemical Shift Referencing website [68], along with tips on how to properly describe the use of all five Methods A–E in a scientific article.

### 2.3. NMR Solvent Signals as Secondary Internal References

**Figure 1:**
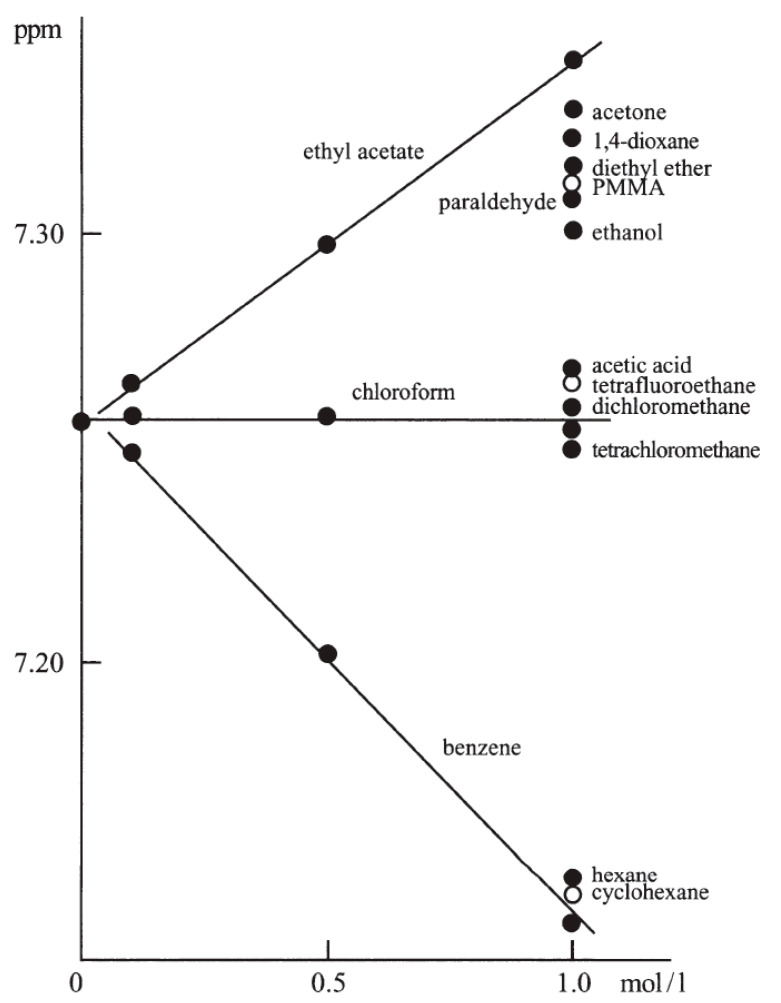
Chemical shifts of the CHCl_3_ line at different concentrations of various analytes studied in CDCl_3_ solution, PMMA = poly(methyl methacrylate) [5]. Reproduced with permission from Springer Nature.

There are many δ_X_ data available in the literature for residual ^1^H and ^13^C signals from deuterated solvents doped with TMS that can be used to indirectly reference the observed NMR signals in Method A. The problem is that *these δ_X_ values differ from each other and can change strongly depending on the type and concentration of the analyte under study* [4,5,90,91]. The influence of these two main factors is shown in Figure 1 [5] for several typical compounds of different types dissolved in CDCl_3_. The impact of the measurement temperature is fairly low.

The results of the search performed for six commonly used TMS-doped NMR solvents are given in Tables S2 and S4 of Supplementary Materials. There is no agreement on the exact δ_X_ values of these internal reference signals in dilute solutions. Therefore, one of the goals of this work was to propose exact δ_X_ values—considering the most reliable data taken from the literature.

These recommended values originally given in the two tables mentioned above are summarized here in Table 2. Unfortunately, these δ_X_ data are, with few exceptions, available without stated uncertainties. Therefore, the values currently proposed were obtained by averaging relatively new data, which appears to be the most reliable. Furthermore, the δ_X_ data that differed significantly from the other data were not taken into account.

All details of the δ_X_ data collected in this search (including some specific examples of ^1^H and ^13^C NMR spectra discussed in the context of using deuterated solvent signals as secondary internal standards) and reported variously directed CIS effects can be found in the Supplementary Materials. In general, binary mixtures of NMR solvents were not considered in this review, except for the three interesting exemplary cases related to refs. [27,92,93] (*vide infra*).

### 2.4. Reference of NMR Spectra Using Method A–Current State

In his book, Jacobsen [92] presents several ^13^C{^1^H} spectra with the CDCl_3_ signal set to 77.00 ppm. However, he recommends the use of Method D implemented in current versions of software on modern NMR instruments [69,70,71,72,73,74,75,76] and some software packages, such as MestReNova [60], because ^13^C signals from deuterated solvents are no longer used for reference in the unified *Ξ* scale (*vide supra*). Finally, he writes: “Old habits die hard, however, and most organic chemists are still using the deuterated solvent peak as a reference” [92] (p. 130). Surprisingly, in this case, the author did not recommend the classical Method B using internal TMS. One might think that only a negligible fraction of NMR spectrometer operators use Method D to record spectra for ^13^C nuclei. Moreover, not all scientists applying NMR spectroscopy in their research use the MestReNova program.

The above citation from ref. [92] perfectly reflects the current state of the practice of internal referencing δ_X_ data using Method A, which is likely to be increasingly applied. It should be emphasized that it was certainly not the intention of the authors of the works [49,51,52,53,54] to introduce this simple procedure into widespread use. They only proposed an easy way to facilitate the identification of common trace impurities in ^1^H and ^13^C NMR spectra, such as silicone grease [94].

Most of the authors of the articles [49,51,52,53,54] describe the variability of δ_H_ data for mobile hydrogen atoms. In some cases, a similar though usually much smaller variation may also apply to non-mobile protons, e.g., CHCl_3_ in CDCl_3_ [4]. In their book, Richards and Hollerton [55] misleadingly wrote that the residual ^1^H signals of CDCl_3_, CD_3_OD, and DMSO-*d*_6_ “are perfectly solid in terms of their shifts.” It is important to remember that all these reported δ_X_ values are only approximate, in part due to the method used to find them [49]. For example, the δ_H_ value for CH_3_CN in the CDCl_3_ solution is reported to be 1.98 [53], 2.00 [95], or even 2.10 ppm [49,51]. Furthermore, to ensure the unambiguous identification of observed resonance signals as specific impurities, a set of ^1^H and ^13^C spectra recorded for the same NMR sample should be analyzed simultaneously [53].

The truth is that, on the Internet and in some books, Method A is explicitly mentioned or even recommended [55]. For example, on the University of Reading (UK) website, you can read that “if TMS is absent from the deuteriated solvent, then the residual protons in the deuteriated solvent can also be used as a secondary reference” (original spelling used) [96]. The identification of such residual lines was discussed in an NMR course for students [97]. Additionally, Leonard et al. [98] mentioned “…the resonance signal for residual CHCl_3_, a peak that is often used as a reference point in ^1^H NMR spectra.” In turn, Armarego and Chai in their very useful book [99] wrote the following about ^13^C signals from deuterated NMR solvents: “In some instances these minor signals have been very useful as internal standards for reporting the chemical shifts of substances, thus avoiding contamination from other added standards, particularly if the samples need to be used for further studies.” However, the authors do not refer to such cases.

Method A (especially its ^1^H variant) is fully applicable to a variety of routine situations, e.g., recording working spectra in optimal reaction conditions or as part of quality control procedures in the chemical or pharmaceutical industry, among others. However, *this simple procedure, in its current form, is usually insufficient for a proper spectroscopic description of all new organic compounds with complex or unexpected molecular structures, especially the isolates from various natural sources*.

In general, it is good practice to use TMS-doped deuterated solvents in Method B to prepare the so-called ‘NMR spectra for publication.’ In fact, internal TMS is “as much an analyte as the actual analyte” [18]. The decisive factor here is the relative insensitivity of δ_H_ for TMS and, to a lesser extent, its δ_C_ values to CIS effects [4,19,20,21,22]. It is usually best to use fresh solvents, e.g., CDCl_3_ stabilized with silver ribbon as a halogen radical scavenger, with a non-minimal amount of TMS, if possible. Therefore, a good alternative is to purchase this compound in small ampoules. This avoids contact with air oxygen and the influence of moisture, which quickly accumulates inside a bottle taken out of the refrigerator. The storage and purification of highly deteriorated CDCl_3_ are discussed in the Supplementary Materials.

In fact, poor storage of CDCl_3_ leads to its acidification. Recently, Teipel et al. [93] showed that the use of this non-fresh solvent in a mixture with CD_3_OD (2:1–1:1, *v*/*v*) to record spectra of various fat extracts (from fish, hen eggs, or coffee) led to some irregular ^1^H NMR signal shifts of these organic materials, which were attributed to the effect of a wet acidic CDCl_3_ present in this binary solvent mixture. According to the authors, there are no reports in the literature regarding ^1^H NMR signal shifts of analytes due to numerous aggressive CDCl_3_ decomposition products, especially DCl/HCl. This literature search confirms this fact. For some comments on storage, numerous possible undesirable side reactions when using ‘old’ acidic CDCl_3_, and its purification, see the short discussion in the Supplementary Materials.

Commonly used deuterated NMR solvents typically containing 0.03% *v*/*v* TMS are only slightly more expensive than their TMS-free counterparts. For those considered here, the price of the former is only 1.05–1.2 times higher. The exception here is CDCl_3_ with silver foil as a stabilizer, which is 1.35 times more expensive than this solvent without a stabilizer. This applies to bottles as well as ampoules. These latter are a bit more expensive. You can also purchase cheaper pure NMR solvents and add TMS directly to the analyzed sample to be dissolved if necessary. A bottle of TMS is generally accessible in most laboratories, as validated by the author of this review’s experiences during his postdoctoral training. Therefore, the issue is probably not the price of solvents with TMS but acquired habits (the long use of Method A) that are difficult to eradicate.

Doping NMR solvents with vapor from a TMS bottle introduced into the sample solution using a Pasteur pipette [100,101] is usually not sufficient to record ^13^C spectra. The authors of [102] used a ’mixed’ A/B method. The ^1^H NMR spectra taken in CDCl_3_ were referenced to internal TMS, but the δ_C_ data were reportedly relative to the solvent signal at 77.0 ppm, although the TMS line is visible in most of the ^13^C{^1^H} spectra provided.

**Figure 2:**
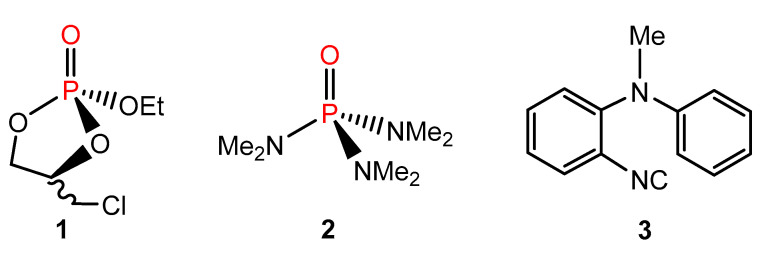
Compounds discussed in refs. [4,103,104].

Finally, it should be mentioned that ^1^H NMR spectra in NMR solvents containing TMS are sometimes quite unexpectedly referenced with respect to residual signals. For example, in [103], the spectrum of product **1** (Figure 2) in CDCl_3_ was related to the CHCl_3_ line at 7.26 ppm [49,51], although TMS was visible at δ_H_ −0.08 ppm (Figure S5). The colors of this spectrum strongly suggest that it was analyzed with MestReNova [60]. The line at −0.01 ppm (instead of 0.07 ppm), which most likely comes from silicone grease [49,51], reinforces this notion. Due to some structural similarities between **1** and hexamethylphosphoramide (HMPA, **2**), which induces a large high-frequency shift of the CHCl_3_ signal [4], it is clear that the δ_H_ values reported in [103] were underestimated by 0.08 ppm.

**Figure 3:**
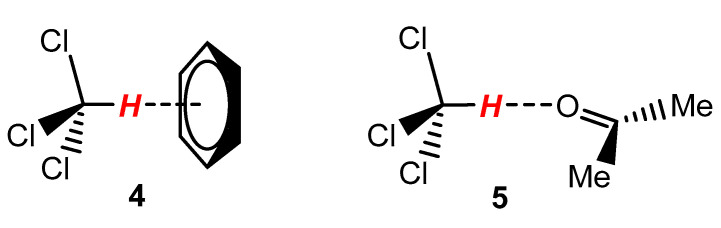
Hydrogen-bonded complexes discussed in refs. [105,106].

A similar case appears in [104], in which “NMR spectra were recorded …. using CDCl_3_ as the solvent. Chemical shifts were reported in parts per million (ppm) using TMS as the internal standard (^1^H NMR: δ = 7.26 ppm, ^13^C NMR: δ = 77.16 ppm).” The above sentence should be regarded as a great mental shortcut that should not appear in a scientific publication. The ^1^H NMR spectrum of the concentrated solution of compound **3**, referenced in this way, indicates the TMS line at ~0.23 ppm (see Figure S6). The associated ^13^C spectrum shows the TMS signal at 0 ppm. Therefore, it can be assumed that, in this case, a corresponding solute–solvent complex was formed (an analog of the well-known molecular system **4** (Figure 3) [105]), which caused a large CIS of the CHCl_3_ line from the usual value of 7.26 ppm [49,51] to ~7.03 ppm. Consequently, the δ_H_ data reported for **3** were overestimated by ~0.23 ppm. Furthermore, it is likely that there were some difficulties in recognizing the weak CHCl_3_ line among the numerous C_ar_H signals due to the high concentration of the analyzed sample.

Apparently, the authors of refs. [103,104] recognized the superiority of a secondary reference standard over a primary standard. The two aforementioned oppositely directed biases due to CIS effects gave a total difference of Δδ_H_ = 0.31 ppm for the CHCl_3_ line. Perhaps this is a partial answer to an important question formulated in the Supplementary Materials about the origin of a large difference in the extreme ‘reference’ δ_H_ values reported by the users of Method A (a range of 7.19 to 7.30 ppm); see Table S2.

In the case of complex **4** (characterized by a large low-frequency CIS effect) [4], interestingly, it was helpful in theoretically verifying the case of an analogously formed acetone–chloroform complex (complex **5**) [106], also showing a very large CIS effect for the CHCl_3_ signal, but in the opposite direction (Figure 1). A brief description of GIAO-DFT-NMR calculations now performed for these two hydrogen-bonded molecular species and a discussion of the results obtained can be found in the computational part of the Supplementary Materials. The high predictive power of the applied theoretical approach was demonstrated.

Chemical shift referencing becomes complicated when binary mixtures of organic solvents must be used, so Method B with a reference material explicitly added to the NMR sample is usually strongly recommended in all such cases. In fact, for this type of tertiary mixture, the analyte has a significant effect on δ_X_s of both the solvents, as well as the secondary internal standard. Thus, in [27], the residual ^1^H line at 7.25 ppm of CDCl_3_, as the main component of the binary solvent mixture, was adopted as an internal reference, although the 2:1 mixture of CDCl_3_ and CD_3_OD was used. As a result, the residual C*H*D_2_OD and CD_3_O*H* protons appeared at 3.09 and 4.25 ppm, respectively, and not at 3.306 and 4.848 ppm—typically observed for pure CD_3_OD doped with TMS (Table 2). Therefore, it could be assumed that the δ_H_ for the CHCl_3_ line in this NMR sample was, in fact, different from the assumed δ_H_ of 7.25 ppm and that the δ_H_ data reported for the analytes studied are subject to some errors.

**Figure 4:**
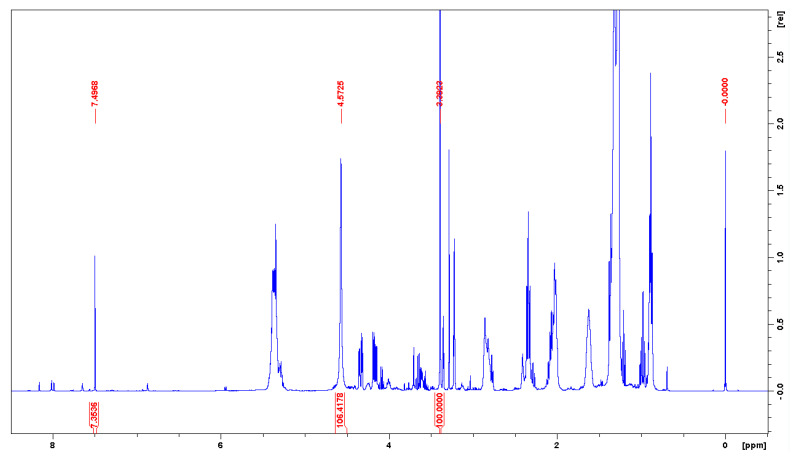
400 Hz ^1^H NMR spectrum of fat extract from trout taken in the mixture of CDCl_3_/CD_3_OD (2:1, *v*/*v*) containing internal TMS [107]. Only important signals are integrated; see text.

The analysis performed in this work concerning a series of ^1^H NMR spectra recently recorded for fat fish extracts [93,107], also taken in the binary mixture (2:1, *v*/*v*) of CDCl_3_ and CD_3_OD, confirmed the previous assumption regarding the δ_H_ data in the article [27]. Figure 4 shows three important signals of the mixture discussed; however, in this specific case, this solvent mixture also contained TMS. These proton signals from C*H*Cl_3_, probably a mixture of *H*_2_O/*H*OD in equilibrium exchange with CD_3_O*H*, and C*H*D_2_OD were found at 7.497 (7.493), 4.572_5_ (4.572), and 3.392 (3.392) ppm, respectively. The above values refer to the initial use of wet and deteriorated CDCl_3_ (Figure 4), while those in parentheses were taken from a very similar ^1^H NMR spectrum (not shown) obtained finally in deacidified CDCl_3_. At this point, it should be mentioned that the signal attributed to the water present in CD_3_OD is observed at 4.87 ppm [49,51,54].

More specifically, both of the spectra mentioned above were taken initially using unpurified (slightly acidic) CDCl_3_ and then using this solvent mitigated by mixing with an aqueous solution of disodium carbonate [93]. This procedure resulted in a 2.5-fold increase in signal intensity at ~4.57 ppm and a minimal high-field shift of the CHCl_3_ line. The CHD_2_OD signal did not change position. Taking into account the differences in δ_H_ values between CHCl_3_ and the other two signals in question, the results are 2.93 and 4.11 ppm. Interestingly, very similar differences in such δ_H_ data apply to the article [27], that is, 3.00 and 4.16 ppm (*vide supra*). Small differences (approximately 6 ppm) may be due to the different types of analytes in the NMR samples discussed. This finding suggests that in the case of ref. [27], the CHCl_3_ line was present at ~7.22 ppm and not 7.25 ppm.

Identical circumstances refer to ^13^C NMR spectra, which are performed very often in CDCl_3_ containing a small amount of DMSO-*d*_6_ to completely dissolve the analyte under study. For example, after adding five drops of it to the indole-2-carboxylic acid sample dissolved in 0.7 mL of CDCl_3_, the signal of the added cosolvent occurred at 38.97 ppm (instead of the typical δ_C_ of 39.46 ppm, see Table 2) when a δ_C_ of 77.00 ppm was assumed for the dominant signal of CDCl_3_ [92] (pp. 171–172). The absence of TMS in the NMR sample makes it impossible to answer the question of whether the presence of the aforementioned analyte changed the position of the ^13^C signal from CDCl_3_.

Another problem arises when δ_D_ data [5,61,63,76] (see Table S5), stored on NMR spectrometer computers and used for automatic lock corrections [61,62], are indirectly used to reference residual ^1^H and ^13^C solvent signals. For example, in a series of ^1^H spectra shown in [108], δ_H_s of 7.26_2_ and 3.34_1_ ppm (found currently after averaging) were given for the residual signals of CDCl_3_ and CD_3_OD, respectively. However, all related ^13^C{^1^H} spectra omit the δ_C_ values of the solvent signals. The absence of these data greatly increases the uncertainty of the published spectral characteristics of the products described. In contrast, in [109], δ_C_ values of ~47.58 and 47.85 ppm obtained from the raw calculation (Table S4, row for the years 2003–2022) were used to calibrate the ^13^C signals of CD_3_OD instead of the typical value of 49.04 ppm. As a result, the δ_C_ data reported for the isolates under study are greatly underestimated.

### 2.5. Proposals Based on Current Needs and Opportunities for NMR Spectrometers

The comparison of δ_X_ data measured for synthesized or isolated organic species with the values reported in the literature for solutions in the same NMR solvents should not be difficult. There may be only minor discrepancies, mainly due to different sample concentrations and/or probe temperatures. Furthermore, all these differences, e.g., of ±0.1 ppm for ^1^H spectra and ±2.0 ppm (or even ±2.5 ppm) [56] for ^13^C spectra, are usually more or less systematic. A certain problem may be the very large difference in the concentrations of the solutions. However, all of these facts are well known among synthetic chemists. In addition to NMR spectra, they usually know the results obtained from the use of other analytical techniques. Thus, there is little doubt that the compounds being compared are identical.

However, for δ_X_ values reported for solutions in the other NMR solvent(s), it will be advisable to use some corrections to compare these data, since the chemical shift of TMS is not zero in all solvents [2,17,18,19,62,89]; δ_H_ = 0 ppm is only in dilute CDCl_3_ solution (*vide supra*). Its δ_H_ has been found to be between −0.8 and 0.2 ppm depending on the solvent; δ_H_ = −0.1277 ppm is for liquid TMS [91]. Generally, the TMS line can vary by more than 1 ppm for ^1^H spectra and 4 or even 5 ppm for ^13^C spectra [19,20,21]. Examples of measured ‘absolute’ solvent-induced shifts of the ^13^C signal of TMS dissolved in CDCl_3_ and CD_3_OD are 0.74 ppm [13] and −0.74 [110] ppm, respectively. The former δ_C_ is equal to the difference between the ‘absolute’ [13,89] and ‘observed’ δ_C_ proposed here for CDCl_3_, Δδ_C_ = 77.75–77.01 = 0.74 (Table 2). The consideration of such δ_C_ data for other NMR solvents without TMS discussed here suggests particularly large TMS–solvent interactions for DMSO-*d*_6_, Δδ_C_ = 40.76–39.46 =1.30 ppm (Table S4). Such changes in δ_C_ values, calculated analogously for (CD_3_)_2_CO and C_6_D_6_, in particular, are much smaller (0.31/0.22 and 0.10 ppm, respectively).

**Figure 5:**
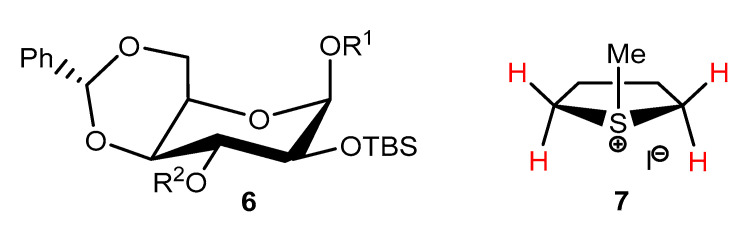
Compounds discussed in refs. [16,113].

Another marginal issue is the frequent complexity of ^1^H NMR spectra of some organic species, which are characterized by strongly coupled spin systems and cannot be analyzed on the basis of first-order assumptions. As a rule, the authors of typical articles do not analyze such spectra and only provide ranges of the observed multiplets. For example, such fragments “4.17–4.12 (m, 1H, H-6b)” and “2.36–1.45 (m, 14H, 5 × CH_2ad_, 4 × CH_ad_)” can be found in the NMR spectral description of a new bicyclic product **6** (Figure 5) [16], despite the fact that its spectrum was measured on a 600-megahertz machine. This example simultaneously demonstrates the use of a current one- and two-decimal standard for reporting δ_C_ and δ_H_ data, respectively [59,111]. Unfortunately, such δ_X_ values, which are usually measured with much higher precision, are rounded off without apparent need at the spectrum analysis stage. MestReNova [60] processes ^1^H NMR spectra in this manner. Accordingly, for more complex spectra, the ranges of multiplets are provided using this program.

Occasionally, you may encounter a qualitatively different issue when the reported molecular structure is questionable. In fact, many structural revisions based on theoretical predictions of the values of NMR parameters have been reported in the literature in recent years. Most of these structural misassignments concern complex organic compounds, especially those isolated from various natural resources (see, e.g., ref. [112]). 

However, it turns out that the incorrect assignment of ^1^H NMR signals can also concern very simple molecules. For example, an erroneous assignment of α-ring protons of the cationic part of ionic system **7** has recently been corrected by DFT-NMR calculations [113]. Furthermore, a full spin analysis of the spectrum of species **7** was carried out via line shape-based iterative refinement and subsequent spectrum simulation. Therefore, in this case, all δ_H_ and *J*_HH_ data were found with a precision of 0.0001 ppm and 0.01 Hz, respectively. Such an analysis is usually the exception rather than the rule (*vide supra*). However, occasionally, by carefully processing raw ^1^H NMR spectra using classical approaches [114,115,116,117], one can easily obtain δ_H_/δ_C_ and *J*_HH_ values with a precision of 0.001/0.01 ppm and 0.01 Hz, respectively [118]. Typically, zero-filling and resolution enhancement with the Lorentzian-to-Gaussian function is performed prior to the Fourier transformation of the spectrum [55,114,115,116,117]; this approach is also available in [60].

It is important to realize that a complete set of the δ_H_ and *J*_HH_ data, pertaining to each complex organic species, including isolates from natural sources, can serve as its unique ^1^H NMR fingerprint [57], especially when these parameters were obtained by ^1^H NMR iterative full spin analysis. Therefore, the Raw Data Initiative highly recommends depositing original properly referenced spectra (or even raw data such as free induction decay (FID) files) for all new natural products for possible structural reanalysis in the future [111,119,120]. Similar suggestions can also be found elsewhere [121]. It should be noted that this approach is fully in accordance with the latest research data policy of the American Chemical Society [122].

Recently, the notion that, in all such cases, the δ_H_s should be provided with at least three but preferably four decimal places when using high-field superconducting magnets to obtain sets of parameters that are suitable as numerical substitutes for current ^1^H NMR spectra is commonplace [111]. The NMReDATA Initiative presented an identical view on the deposition of computer records of such spectra for all new complex synthetic and natural organic systems [123]. Publishing δ_H_ values with a precision of 0001 ppm is recommended.

The use of the δ_H_ and δ_C_ data with higher precision than is currently suggested is particularly important for all species with high C/H ratios or with proton deficiency and many heteroatoms, or more generally, for organic systems that provide a small number of δ_X_ data. A suitable method to use in such cases is the multinuclear method [124], which includes a non-1:1 correlation between theoretical predictions and experimental δ_X_ data. This approach uses δ_H_ values multiplied by an adjustable factor *n*, for example*, n* = 10. In this case, the δ_H_/δ_C_ data measured for the same NMR samples containing TMS and reported to the nearest 0.001/0.01 ppm are mandatory.

An application of ^13^C signals from deuterated solvents to standardize the δ_C_ values is justified in part because the range of typical δ_C_ data is approximately 20 times larger than the δ_H_ range that suffers from a much lower spectral dispersion of δ_H_ data. For this reason, except for the cases of some specific isomeric systems, the reported δ_C_ values rounded to one decimal place are recommended [59].

However, given the large inaccuracies in the δ_X_ values used in Method A (see the Supplementary Materials for all the details) and the relatively high dependence of δ_C_ on the type/concentration of the analyte studied (*vide supra*), it is crucial that the adopted ‘reference’ δ_X_ data are given accurately in publications. Therefore, it should be mandatory that these δ_H_ and δ_C_ values should be provided within 0.001 and 0.01 ppm because this affects the precision of δ_X_ data reported. The authors of [125] provide a positive example here. Unfortunately, in many of the articles cited above and recently published, such δ_X_ data do not appear at all [23,29,30,58,126,127,128]. It should be stressed that the aforementioned precision is achieved seamlessly on all 400-MHz NMR machines with an inherently lower digital resolution.

Finally, it should be noted that Burns and Reynolds in their book [129] recommend that δ_H_ and δ_C_ values for all new organic species or isolates should be given in three and two decimal places, respectively, with the last place subscripted to indicate uncertainty due to sample concentration and/or probe temperature. Furthermore, they considered Methods A and B equivalent because, in their opinion, residual ^1^H and ^13^C solvent signals have similar uncertainties when used in place of TMS. 

Recent findings by Guzman and Hoye [4] did not confirm the aforementioned view for ^1^H NMR spectra in CDCl_3_. In addition, most of the cases discussed in this work related to the standardization of ^13^C NMR spectra using solvent signals do not seem to agree with the above opinion [129]. However, only a more detailed investigation of this issue can provide a reliable view of the matter.

## 3. Summary and Conclusions

This short review article sketches the most likely ‘birth’ of Method A for popular, simple but not perfect referencing of NMR spectra, involving their indirect standardization using residual ^1^H and ^13^C signals from deuterated solvents as secondary reference points on the chemical shift scale. Undoubtedly, the inclusion of this procedure in the arsenal of various NMR spectroscopy methods had to be preceded by the introduction of pulsed Fourier-transform instruments in the early 1970s. All documents and information found in the literature review indicated that Method A became widespread as a result of the initiative of NMR users, especially synthetic organic chemists. The obvious catalysts for this process were the acceptance of TMS as an internal zero-point standard for ^13^C spectra and the common availability of ‘reference’ δ_H_ and then δ_C_ data as secondary internal standards. The increasingly popular use of this method to record and report ^1^H and ^13^C NMR spectral data is briefly described here. 

Regarding the new results obtained now, all ‘reference’ δ_X_ data found in the literature concerning six common NMR solvents doped with TMS were considered and can be found in the Supplementary Materials (Tables S2 and S4). This allowed the proposal of their ‘best’ values for use in Method A, which are summarized in Table 2. The large variability of these δ_X_ data was also analyzed in detail. The influence of the concentration and type of the analyte tested is greatest, while the directions and magnitudes of such CIS result from various solvent–analyte interactions that occurred in a particular case. The large δ_H_ changes in the position of the CHCl_3_ line observed in the presence of acetone or benzene in TMS-doped CDCl_3_ [4,5] were reproduced very well in the current DFT-NMR calculations carried out for hydrogen-bonded complexes **4** and **5** formed in this solvent (see Supplementary Materials). Therefore, it is clear that dilute solutions should generally be applied when Method A is used to minimize all such CIS effects for real NMR samples as much as possible.

As for the current state of Method A, its practical application is shown here in the convention of good and numerous bad exemplary cases. Statistically, the use of this simple method is generally acceptable, but there is still plenty of room for improvement. Focusing only on the procedures for post-processing the recorded NMR spectra, it is possible to mention some basic problems and pitfalls associated with Method A, which are mainly due to the mistakes of the researchers themselves as end users.

In particular, this includes the adoption of inappropriate ‘reference’ δ_X_ data, which leads to an over- or under-estimation of δ_X_ data finally reported. Similar problems always occur when mixtures of NMR solvents are used. In such cases, it is necessary to use Method B (*vide supra*). The adopted secondary ‘reference’ data or their sources from the literature are often not given at all or only partially, which should not be the case. This is especially true for solvent signals in published copies of NMR spectra, often without providing their δ_X_ values. Similar problems arise, for example, when using MestReNova [60] without knowing the limitations of Method A. 

The recommended use of the above program [60] leads to the generation of ^1^H NMR spectral data in the form of a list of signals for which the number of protons and possibly *J*_HH_ data are given. Sometimes, only the ranges of multiplets are given for certain spectrum regions (*vide supra*). In general, ^1^H/^13^C spectra are readily available for inclusion in a publication after analysis, but their precision strongly depends on the researcher as the end user. He must be fully aware of the limitations of Method A. The lack of a primary TMS signal makes it difficult to use. As a result, the reported δ_X_ data may be subject to some errors and the published spectra may lack important reference values for the NMR solvent signals adopted in the analysis performed. Unfortunately, the belief in this type of user-friendly software is often too high. Different examples of such issues can be found in the Search Results and Discussion section.

It is easy to list numerous drawbacks or pitfalls as well as only a few benefits of using Method A. The first is mainly due to the absence of generally accepted rules for its application. In particular, this includes the lack of a formal obligation to provide the exact δ_X_ values of the reference ^1^H and ^13^C solvent signals used to report spectral data for all new organic species. The second issue is due to possible signal overlap. In fact, there may be problems with the spectra of aromatics referenced by applying the residual ^1^H lines from CDCl_3_ or C_6_D_6_ (see above for an exemplary case of species **3**). The use of TMS or cubane as an internal reference can be useful in all such situations (for the latter, see Table S3 and the related discussion). However, the main problem is the occurrence of numerous CIS effects, especially when using quite concentrated NMR samples. These effects can often lead to significant changes in the δ_X_ values adopted for the secondary references in question to lower or higher frequencies, depending on the type of analyte.

It should also be highlighted that, in the absence of TMS in the NMR sample, Method A is used indirectly in Method C. More importantly, it is also applied in an IUPAC-recommended Method D used to reference spectra involving some NMR-active nuclei that are different from ^13^C (*vide supra*). Strictly, ^13^C spectra can also be standardized in this way, but solvent signals are generally used for this purpose. Therefore, the correct referencing of the initial ^1^H spectra using Method A is decisive in all these cases.

In view of all the foregoing facts, it should be mandatory to give the reference δ_H_ and δ_C_ data adopted in Method A with a precision of 0.001 and 0.01 ppm, respectively, and report the spectral data for the products or isolates with the same precision. If there is any doubt about the rounding of some values (up or down), it is recommended that one should insert the digit 5 in the additional decimal position given in the subscript. To avoid prolonging the main text of the publication, the δ_X_ data with such precision has been included in the Supplementary Materials section. The above precision requests may be seen to be somewhat excessive, especially for organic chemists, who usually have additional experimental data at their disposal when identifying the synthesized products. However, as noted earlier, these requirements particularly apply to all new complex or unexpected structures. In such cases, identical requirements should also be applied to the use of Methods B–D.

One of the few advantages of Method A (used as mentioned above) is that its application avoids contamination of the samples being analyzed by TMS. It is likely that its use could be useful in some cases where the NMR samples under study need to be used for further special studies, as suggested in ref. [99]. Indisputably, silyl derivatives possessing signals with δ_H_s very close to 0 ppm may be obvious targets for this approach (*vide supra*). Using the classical iteration of Method B with an internal TMS can be difficult in such cases. This may be especially true for spectra recorded on older 200-MHz spectrometers.

According to the results of this literature review, there is a significant discrepancy of 1.7 and even 1.9 ppm in the δ_C_ values for DMSO-*d*_6_ and CDCl_3_, respectively, applied by the users of Method A (Table S4, two lines at the bottom); these results are in line with the large uncertainties in δ_C_ values for these solvents reported in ref. [49]. Smaller differences in the δ_C_s concern CD_3_OD and especially CD_3_CN. One can only assume that the sources of the aforementioned differences in δ_C_ data are the CIS effects resulting from the presence of various analytes in the NMR samples studied. These findings suggest the need for a systematic investigation of ^13^C{^1^H} spectra recorded in all solvents discussed here for analytes of different types, simultaneously using ^13^C signals from TMS and NMR solvent, analogously to the study performed for ^1^H NMR spectra in CDCl_3_ [4]. A similar investigation carried out more than two decades ago produced unsatisfactory results [56].

The careful and correct application of each of the four Methods A–D to fairly dilute solutions of different organic species in deuterated organic solvents should, in principle, lead to very similar δ_H_ and δ_C_ data for analytes of different types. To the author’s knowledge, to date, no comparative studies similar to those performed for ^19^F NMR spectra [12] have been carried out.

One might be tempted to conclude that the daily practice of NMR spectroscopy has, so far, outgrown the recommendations of the IUPAC and other similar regulations that have not yet taken into account the widespread use of Method A, in which experimentally measured δ_X_ data are related to internal TMS at 0 ppm, albeit indirectly. 

Therefore, it is probably time for an in-depth discussion and formulation of some undoubtedly needed rules for the best possible use of Method A. Its application will probably expand due to the increasing frequency of milligram-scale chemical syntheses and the growing use of benchtop NMR instruments [7,130]. Indeed, by using the latter relatively inexpensive devices, residual ^1^H signals from deuterated solvents are frequently applied [130]. In general, a proper routine use of Method A for the referencing of ^1^H and ^13^C spectra with all of the above requirements seems to have great potential, especially in the context of the increasingly widespread use of cryogenically cooled probes of classical NMR spectrometers, which allows for the analysis of very dilute solutions.

However, for all newly synthesized or isolated organic species, especially those with complex or unexpected molecular structures, the use of TMS in Method B is strongly recommended to ensure the precise ^1^H and ^13^C NMR spectroscopic characterization of the organic systems mentioned above.

## Figures and Tables

**Table 1:** Five standard ^1^H and ^13^C NMR chemical shift reference methods *^a^*.

Method	Description	Notes
A	Residual ^1^H and ^13^C signals from NMR solvents are used as internal standards–two variants (^1^H and ^13^C)	A simplified, formally unregulated but widely applied method, the use of which is discussed in detail here
B	By using internal references (mainly TMS)	Standard approach–a codex of chemistry (TMS)
C	Use ^2^H known lock frequencies of the NMR solvents	Default method on all modern NMR spectrometers
D	An accurate IUPAC-recommended general scheme for indirect referencing based on *Ξ* values	Used for all NMR-observable nuclei, but only very rarely routinely for ^1^H and ^13^C
E	Use of external standards (typically) in coaxial tubes	Used most often for the ^11^B, ^15^N, ^19^F, ^31^P nuclei, etc.

*^a^* By using standard cylindrical NMR tubes.

**Table 2:** Recommended δ_X_ values for ^1^H (residual) and ^13^C signals of six common NMR solvents measured vs. internal TMS, ppm.

	CDCl_3_	(CD_3_)_2_CO		(CD_3_)_2_SO	C_6_D_6_	CD_3_CN		CD_3_OD	
δ_H_	7.260	2.053		2.502	7.156	1.939		3.306	4.848 *^a,b^*
δ_C_	77.01	29.83	206.15 *^b^*	39.46	128.03	1.36	118.36	49.04	

*^a^* The CD_3_O*H* signal. *^b^* The application of this signal in Method A is usually not recommended due to the high variability of its δ_X_ value (see Supplementary Materials).

## Data Availability

Not applicable.

## Supplementary Materials

The following supporting information can be downloaded at: https://www.mdpi.com/article/10.3390/molecules28114369/s1, The experimental ‘reference’ δ_H_ and δ_C_ values from the literature for six deuterated solvents without TMS, including MSD data (Table S1), a continuation of the subsection on solvent signals as secondary NMR standards, including different CIS effects, a discussion of cubane and cyclohexane as internal references (Table S3), two new subsections on ^1^H and ^13^C spectra (Tables S2 and S4 covering 45 sets of δ_X_ data) including δ_D_ values for six solvents considered (Table S5), stacked ^1^H NMR spectra from the ‘titration’ of residual incompletely deuterated components of the four deuterated solvents with tetra-*n*-butylammonium chloride as a titrant (Figures S1–S4) and two cases of not correctly analyzed ^1^H NMR spectra (Figures S5 and S6); an additional section with description and discussion of the results of GIAO-DFT-NMR calculations performed now for hydrogen-bonded complexes **4** and **5** (Tables S6 and S7, Figure S7); [131,132,133,134,135,136,137,138,139,140,141,142,143,144,145,146,147,148,149,150,151,152,153,154,155,156,157,158,159,160,161,162,163,164,165,166,167,168,169,170,171,172,173,174,175,176,177,178,179,180,181,182,183,184,185,186] references to the Supplementary Materials part (PDF).
