# Supplementary material for: On the Use of Deuterated Organic Solvents without TMS to Report 1H/13C NMR Spectral Data of Organic Compounds: Current State of the Method, Its Pitfalls and Benefits, and Related Issues
Source: Molecules. 2023 May 26;28(11):4369. doi: 10.3390/molecules28114369 (PMC10254718; doi:10.3390/molecules28114369)
Supplement: Supplementary file 1 [file molecules-28-04369-s001.zip › Supplementary Materials (revisedB) - Nazarski.pdf]

Supplementary Materials

for

On the Use of Deuterated Organic Solvents without TMS to Report ^{1}H/^{13}C NMR Spectral Data of Organic Compounds: Current State of the Method, Its Pitfalls and Benefits, and Related Issues

by

Ryszard B. Nazarski*

Theoretical and Structural Chemistry Group, Department of Physical Chemistry, Faculty of Chemistry, University of Lodz, 163/165 Pomorska, 90-236 Łódź, Poland

orcid.org/0000-0001-5063-3912

*E-mail: *ryszard.nazarski@chemia.uni.lodz.pl*

Table of Content:

Experimental data from the literature and their analysis S2

Table S1. Deuterated NMR solvents – Handy Reference Data (from MSD Isotopes) S2

NMR solvent signals as secondary internal references – supplementary part S3

Table S2. Compilation of δ_Hs reported for residual ^{1}H signals of six common NMR solvents S3

Selected cases relating to the ^{1}H NMR spectra S4

Table S3. Compilation of the δ_x data for the signals of cubane and cyclohexane S4

Figure S1. ^{1}H NMR titration spectra obtained using salt **2** (TBACl) in acetone-d_6 S6

Figure S2. ^{1}H NMR titration spectra obtained using salt **2** (TBACl) in DMSO-d_6 S6

Figure S3. ^{1}H NMR titration spectra obtained using salt **2** (TBACl) in acetonitrile-d_3 S7

Figure S4. ^{1}H NMR titration spectra obtained using salt **2** (TBACl) in methanol-d_4 S7

Selected cases relating to the ^{13}C NMR spectra S8

Table S4. Compilation of δ_Cs reported for ^{13}C signals of six common NMR solvents S8

Table S5. Compilation of δ_Ds reported for ^{2}H signals of six common NMR solvents S9

Two exemplary cases of an incorrect analysis of ^{1}H NMR spectra from the literature S10

Figure S5. 400 MHz ^{1}H NMR spectrum of compound **1** from the main text S10

Figure S6. 400 MHz ^{1}H NMR spectrum of compound **3** from the main text S11

Computational results obtained in this work S12

Table S6. The δ_X data predicted for species **7** and **8** as well as for TMS and $CHCl_3$ S12

Table S7. Optimized geometries of complexes **7** and **8** S13

Figure S7. The 3D shapes of complexes **7** and **8** S13

References for Supplementary Materials S14

Experimental data from the literature and their analysis

Table S1. Reproduction from Ref. [1] with permission from University Science Books

TABLE 3[a]

DEUTERATED NMR SOLVENTS-HANDY REFERENCE DATA

Compound<br>Mol. Wt.	d_4^{20}	m.p.*	b.p.*	δ_H (mult)[b]	J_{HD}	δ_C (mult)[b]	J_{CD} (J_{CF})
Acetic Acid-d_4<br>64.078	1.12	17	118	11.53 (1)<br>2.03 (5)	 2	178.4 (br)<br>20.0 (7)	 20
Acetone-d_6<br>64.117	0.87	-94	57	 2.04 (5)	 2.2	206.0 (13)<br>29.8 (7)	0.9<br>20
Acetonitrile-d_3<br>44.071	0.84	-45	82	 1.93 (5)	 2.5	118.2 (br)<br>1.3 (7)	 21
Benzene-d_6<br>84.152	0.95	5	80	7.15 (br)		128.0 (3)	24
Chloroform-d<br>120.384	1.50	-64	62	7.24 (1)		77.0 (3)	32
Cyclohexane-d_{12}<br>96.236	0.89	6	81	1.38 (br)		26.4 (5)	19
Deuterium Oxide<br>20.028	1.11	3.8	101.4	4.63 (DSS)<br>4.67 (TSP)			
1,2-Dichloroethane-d_4<br>102.985	1.25	-40	84	3.72 (br)		43.6 (5)	23.5
Diethyl-d_{10} Ether<br>84.185	0.82	-116	35	3.34 (m)<br>1.07 (m)		65.3 (5)<br>14.5 (7)	21<br>19
Diglyme-d_{14}<br>148.263	0.95	-68	162	3.49 (br)<br>3.40 (br)<br>3.22 (5)	 <br>1.5	70.7 (5)<br>70.0 (5)<br>57.7 (7)	21<br>21<br>21
Dimethylformamide-d_7<br>80.138	1.04	-61	153	8.01 (br)<br>2.91 (5)<br>2.74 (5)	 2<br>2	162.7 (3)<br>35.2 (7)<br>30.1 (7)	30<br>21<br>21
Dimethyl-d_6 Sulphoxide<br>84.170	1.18	18	189	2.49 (5)	1.7	39.5 (7)	21
p-Dioxane-d_8<br>96.156	1.13	12	101	3.53 (m)		66.5 (5)	22
Ethyl Alcohol-d_6 (anh)<br>52.106	0.91	<-130	79	5.19 (1)<br>3.55 (br)<br>1.11 (m)		 56.8 (5)<br>17.2 (7)	 22<br>19
Glyme-d_{10}<br>100.184	0.86	-58	83	3.40 (m)<br>3.22 (5)	 1.6	71.7 (5)<br>57.8 (7)	21<br>21
Hexafluoroacetone Deuterate<br>198.067	1.71	21		5.26 (1)		122.5 (4)<br>92.9 (7)	(287)<br>(34.5)
HMPT-d_{18}<br>197.314	1.14	7	106 (11)	2.53 (2 x 5)	2 (9.5)	35.8 (7)	21
Methyl Alcohol-d_4<br>36.067	0.89	-98	65	4.78 (1)<br>3.30 (5)	 1.7	49.0 (7)	21.5
Methylene Chloride-d_2<br>86.945	1.35	-95	40	5.32 (3)	1	53.8 (5)	27
Nitrobenzene-d_5<br>128.143	1.25	6	211	 8.11 (br)<br>7.67 (br)<br>7.50 (br)		148.6 (1)<br>134.8 (3)<br>129.5 (3)<br>123.5 (3)	 24.5 (p)<br>25<br>26
Nitromethane-d_3<br>64.059	1.20	-29	101	4.33 (5)	2	62.8 (7)	22
isoPropyl Alcohol-d_8<br>68.146	0.90	-86	83	5.12 (1)<br>3.89 (br)<br>1.10 (br)		 62.9 (3)<br>24.2 (7)	 21.5<br>19
Pyridine-d_5<br>84.133	1.05	-42	116	8.71 (br)<br>7.55 (br)<br>7.19 (br)		149.9 (3)<br>135.5 (3)<br>123.5 (3)	27.5<br>24.5 (γ)<br>25
Tetrahydrofuran-d_8<br>80.157	0.99	-109	66	3.58 (br)<br>1.73 (br)		67.4 (5)<br>25.3 (br)	22<br>20.5
Toluene-d_8<br>100.191	0.94	-95	111	 7.09 (m)<br>7.00 (br)<br>6.98 (m)<br>2.09 (5)	 <br><br><br>2.3	137.5 (1)<br>128.9 (3)<br>128.0 (3)<br>125.2 (3)<br>20.4 (7)	 23<br>24<br>24 (p)<br>19
Trifluoroacetic Acid-d[c]<br>115.030	1.50	-15	72	11.50 (1)		164.2 (4)<br>116.6 (4)	(44)<br>(283)
2,2,2-Trifluoroethyl Alcohol-d_3[e]<br>103.059	1.45	-44	75	5.02 (1)<br>3.88 (4 x 3)	 2 (9)	126.3 (4)<br>61.5 (4 x 5)	(277)<br>22 (36)

*Melting and boiling points (in °C) are those of the corresponding light compound (except for D_2O) and are intended only to indicate the useful liquid range of the materials.

[b] ^1H (of the residual protons) and ^{13}C spectra were determined on HA-100 and XL-100-15 spectrometers, respectively, for the same sample of each solvent containing 5% TMS (v/v). The chemical shifts are in ppm relative to TMS; the coupling constants are in Hz. (Since deuterium has a spin of 1, triplets arising from coupling to deuterium have the intensity ratio of 1:1:1, etc.) The multiplicity br indicates a broad peak without resolvable fine structure, while m denotes one with fine structure. It should be noted that the chemical shifts, in particular, can be dependent on solute, concentration and temperature.

[c] δ_F($CFCl_3$) 82.6 (1) [d] δ_F($CFCl_3$) 76.2 (1) [e] δ_F($CFCl_3$) 77.8 (5), J_{FD} 1.2 all determined on an HA-100 spectrometer.

P.O. BOX 2951 TERMINAL ANNEX
LOS ANGELES, CA 90051
213 723-9521
800 325-9034
4545 OLEATHA AVE.
ST. LOUIS, MO 63116
314 353-7000
MERCK & CO., Inc.
ISOTOPES
P.O. BOX 899
POINTE CLAIRE-DORVAL
QUEBEC H9R 4P7
514 697-2823
ISOTOPES

[a]Reprinted with permission from MSD Isotopes

NMR solvent signals as secondary internal references – supplementary part

Several different tables can be found in the literature with the 'reference' δ_X data on residual ^{1}H and ^{13}C signals from deuterated NMR solvents doped with TMS, which can be used to indirectly refer analyte signals to TMS in Method A. As already written in the main text, the problem is that the reported values of these δ_X data differ from each other and can also change strongly depending on the type and concentration of the analyte under study. The impact of the measurement temperature is usually less [2–5]. This situation for ^{1}H NMR spectra recorded in $CDCl_3$ is very well illustrated in Figure 1 of the main text.

The results of a comprehensive search conducted for the six most popular organic NMR solvents are given in Tables S2 and S4. All the δ_Xs gathered are taken from original articles, books, booklets, or other sources. Generally, only δ_X data reported for full sets of TMS-doped solvents are included. Some exceptions are the δ_Cs found by Levy [6,7] and the very important set of four 'absolute' δ_C values for NMR solvents without TMS measured under MAS conditions [8] (Table S4, data in italics).

The bold font indicates the δ_Xs that appear to be the most reliable. There is no consensus on their values, and one of the goals of this work was to propose them. Unfortunately, these data are, with few exceptions, available without uncertainties. Currently, proposed δ_Xs are given in red, underlined, and bolded in Tables S2 and S4. They were obtained by averaging relatively new values and being trustworthy. In general, large outliers were not taken into account. However, for real solutions, such δ_X data tend to deviate more or less due to the CIS effects already mentioned in the main text. Many additional cases of CISs are also discussed below**.** Therefore, using Method A, it is not certain that these δ_X values will be appropriate to reference the individual NMR samples under study. *All these δ_X data are only the expected chemical shifts for the respective solvent signals*.

Great care has been taken to maintain the chronological order in Tables S2 and S4. Consequently, the oldest data are presented first. This seems quite significant since these δ_X values have often changed over time, *e.g.*, in the newer editions of some books. Such a compilation can be very helpful to potential readers of works in which the adopted δ_X data or their sources are not given. This article deals only with six organic NMR solvents, but the δ_Xs for other deuterated solvents can be found in the references. It should be noted that the exact values of the δ_X data discussed here were also determined against liquid TMS [9,10].

Table S2. Compilation of δ_H values reported for residual ^{1}H signals of six common NMR solvents, ppm (*vs.* internal TMS).

$CDCl_3$	$(CD_3)_2CO$	$(CD_3)_2SO$	C_6D_6	CD_3CN	CD_3OD	CD_3O*H*[a]	Year	References
7.25	2.05	2.50	7.20	1.95	3.35	4.84	≤ 1967	[11]
7.25	2.07	2.50	7.24	1.96	3.34	4.1	1972	[12]
7.24	2.04	2.49	7.15	1.93	3.30	4.78	≤ 1978-2011	[1,13-19]
7.25	2.05	2.5	7.15	1.95	3.35	4.8	1979	[20]
7.26	2.05	2.5	7.16	1.93	3.31	4.79	1988	[21]
7.24	2.05	2.50	7.16	1.94	3.31	4.78	≥ 1993	[22,23]
7.26	2.05	2.50	7.16	1.94	3.31	4.87	≥ 1997-2016	[24-26]
7.263	2.052	2.503	7.155	1.940	-	-	1999-2010	[27]
7.24	2.04	2.49	7.12	1.93	3.30	4.78	2003-2011	[28,29]
7.26	2.04	2.50	7.16	1.94	3.30	4.85	2003	[30]
**7.258**	**2.051**	**2.500**	**7.156**	**1.938**	**3.306**	-	2004	[2]
7.27	2.05	2.50	7.16	1.93	3.31	-	2005	[31]
7.261	2.053	2.504	-	1.940	3.312	4.867	2006	[32]
7.27	2.05	2.50	7.16	1.94	3.31	4.87	2009-2016	[33,34]
7.26	2.05	2.50	7.16	1.94	3.31	4.84	2009-2022	[35,36]
**7.258**	**2.052**	**2.501**	**7.157**	**1.938**	**3.306**	**4.849**	2010	[37]
7.27	-	2.50	7.27	-	3.30	-	2011	[38]
7.26	2.04	2.49	7.15	1.93	3.30	4.78	2015	[39]
7.24	2.06	2.50	7.16	-	-	-	2016	[40]
7.24	2.04	2.49	7.15	1.93	3.30	4.89	2022	[41]
**7.260_5**	**2.054_5**	**2.501**	**7.157**	**1.940**	**3.306**	**4.847**	2022	[5]
**7.261**	**2.053**	**2.504**	**7.154**	**1.940**	**3.306**	**4.847**	2022	[42]
**7.262**	**2.053**	**2.502**	**7.157_5**	**1.939**	**3.307**	**4.848**	2022	[43]
**7.260**	**2.053**	**2.502**	**7.156**	**1.939**	**3.306**	**4.848**		**Recommended values**
[7.19]	[2.00]	[2.47]	-	-	-	-	2013-2020	For details, see text
[7.30]	[2.09]	[2.54]	-	-	-	-	1998-2022	For details, see text

[a] This δ_H may vary greatly depending on the nature of the analyte and the probe temperature [20]; recognized [24–26] as an H_2O signal.

The extreme δ_X values collected, which are firmly outside the range of the 'reference' data, are listed in square brackets in the two lowest rows of Tables S2 and S4. All of these outliers come from papers whose authors used Method A. The data reported below strongly suggest that all of these extreme values were most likely due to the high- or low-field CISs of respective reference signals from the NMR solvents used.

Selected cases relating to the ^{1}H spectra

A closer look at the oldest δ_H data tabulated for C_6D_6 and CD_3OD leads us to believe that they were found for concentrated solutions, which were standard at the time. In turn, the newer values [13] taken from spectra recorded at a frequency of 100 MHz are, relative to the current data, underestimated by 0.01 ppm and even 0.02 ppm for the $CHCl_3$ line (δ_H of 7.24 ppm). Of particular notation are the δ_H data found in the presence of cubane (**1**) as an analyte [37]. For only three of six cases, they increased by 0.001 ppm relative to δ_H of similar precision found for pure TMS-doped solvents [2]. Notable is the poor quality of δ_H data provided in the encyclopedic book [28,29].

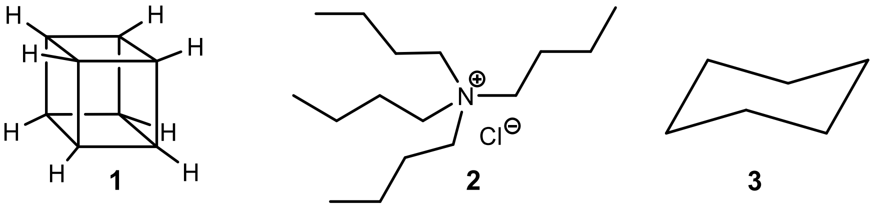

1 **2** **3**

In the following, interesting results for various CISs are discussed as examples of the impact of the solute type on the direction of changes in the δ_H data for the 'residual' solvent signals. Therefore, tetra-*n*-butylammonium chloride (TBACl, **2**) dissolved in five deuterated solvents was used as an analyte. This ionic system, which provides so-called naked chloride anions in the reaction medium, is often used in organic synthesis as an initiator of some chemical processes [44]. Similar δ_H data were obtained for **2** in parallel control measurements done in the same NMR solvents with TMS as an external standard in Method E [45–49].

It may be mentioned here that the signal overlap issue similar to that for TMS and silyl derivatives potentially also exists for using residual C*H*Cl_3 or C_6D_5*H* signals to characterize aromatic systems. Both problems were briefly discussed in the main text (regarding aromatics; see the case of compound **3** discussed there). A certain solution may be to use internal TMS or even compound **1** (Method B). The δ_X data for using **1** in six TMS-doped solvents [37] are collected in Table S3 together with the data for cyclohexane (**3**), which has often been used as a reliable internal standard [50–52]. An inspection of the table, more specifically the differences δ_X(**1**)–δ_X(**3**), reveals that the degree of interaction of NMR solvents with the two above analytes is quite similar.

Table S3. Compilation of δ_C data for the signals of cubane (1) and cyclohexane (**3**) in six common NMR solvents, ppm (*versus* internal TMS, at around 25 ^{o}C) [a]

Analyte / *differences*	$CDCl_3$	$(CD_3)_2CO$	$(CD_3)_2SO$	C_6D_6	CD_3CN	CD_3OD	References
			**Proton Shifts**				
**1**	4.03_2	4.03_7	4.02_7	3.94_7	4.04_5	4.05_0	[37]
**3**	1.43	1.43	1.40	1.40	1.44	1.45	[24-26]
*δ_H(**1**) – δ_H(**3**)*[a]	*2.60*	*2.61*	*[2.63]*	*[2.55]*	*2.60_5*	*2.60*	This work[b]
			**Carbon Shifts**				
**1**	47.74	48.35	46.62	48.05	48.56	48.98	[37]
**3**	26.94	27.51	26.33	27.23	27.63	27.96	[24-26]
*δ_C(**1**) – δ_C(**3**)*[a]	*20.80*	*20.84*	*[20.29]*	*20.82*	*20.93*	*[21.02]*	This work[b]

[a] The extreme values of these differences are given in square brackets. [b] For a brief discussion of results, *see some cases concerning the ^{1}H spectra* subsection.

Chloroform-d. The δ_H 7.24 ppm was recorded on a 200 MHz spectrometer [22]. This value was also quoted in the handbook [40] as measured at 600 MHz. The problem of 7.24 versus 7.26 ppm for the $CHCl_3$ line has already been discussed [53,54]. Initially, the δ_H of 7.2400 ppm was adopted [53], but in a later paper [54], however, 7.2600 ppm was used. The δ_H 7.261 ppm was recently determined for this component of $CDCl_3$ (pure or doped with TMS) under three different measurement conditions [55]. In turn, an overestimated value of 7.27 ppm [38,56] was most likely due to the use of a concentrated solution. The extreme data found are 7.19 [57] and 7.30 [58,59] ppm.

Generally, the analyte-dependent shifts of the $CHCl_3$ line are within ± 0.01 ppm when the analyte concentration is below 0.1 mol/L [2]. However, larger CISs, from δ_H 7.26 to 7.14 and 7.33 ppm, respectively, were found for a 1 mol/L solution of benzene (**4**) and acetone (**5**); see Figure 1 (taken from Ref. [2]) in the main text. The δ_H change from 7.260 to 7.488 ppm was also found for a saturated solution of ammonium salt **2**; a linear relationship (R^2 = 0.999) was established between the concentration of **2** and δ_H for $CHCl_3$ [45,46]. Even larger CISs were found [4] to be 6.84, 7.87, and even 9.00 ppm for 4 mol/L solutions of compounds **4**, **5**, and HMPA (**6**), respectively.

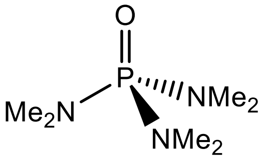

6

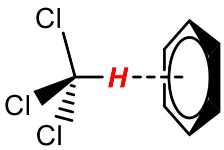

7

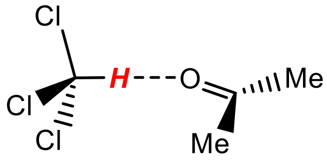

8

With a change in the role of the two aforementioned systems **4** and **5**, the CISs are much more pronounced; the $CHCl_3$ line was found at 6.15 and 8.02 ppm in the C_6D_6 and acetone-d_6 solutions, respectively [24]. Both of these experimental trends were well reproduced for the formed 1:1 hydrogen-bonded molecular complexes **7** [60] and **8** [61] in typical [62–66] density functional theory (DFT) NMR predictions carried out in this work. All details and a brief discussion of the results obtained (Tables S6/S7 and Figure S7) can be found in the *computational results obtained in this work* section. In turn, an even greater CIS of the $CHCl_3$ line was reported for the DMSO-d_6 solution (δ_H 8.32 ppm) [24].

The content of the lighter isotopologue of $CDCl_3$ usually varies within a certain range, which may be due to different manufacturing, packing, transportation, and storage conditions [67]. It should be emphasized that non-stabilized $CDCl_3$, unlike all other NMR solvents discussed here, is a time-sensitive compound and should be stored in the refrigerator (–5 to 5 °C) away from light and moisture [22,68]. Its prolonged and/or poor storage (especially after opening the bottle) causes gradual acidification through partial oxidative photodegradation to trace amounts of DCl/HCl and other decomposition products, and moisture accelerates this process [69–71]. Such a wet unpurified (acidic) $CDCl_3$ can catalyze undesirable reactions [71–77]. These transformations can even occur *in situ* in an NMR tube [74]. Its standard purification usually involves passing it through a chromatographic column, even using a Pasteur pipette, filled with freshly activated alkaline aluminium oxide (alumina) [68,72,76,78].

***Acetone-d*₆**. The deviations from the 'reference' position found (2.00 [59,79] and 2.09 [80] ppm) are similar to those of $CDCl_3$. Unlike what was reported for CD_3Cl, the ^{1}H line of CD_3CD_2HCO undergoes a large up-field CIS in the presence of chloride ions. The largest change from 2.059 to 1.786 ppm was observed for a saturated solution of ammonium salt **2**, see Figure S1 [47].

DMSO-d_6. Changes in the *'reference' value* are rather small (up to 2.47 [81] or 2.54 [82] ppm). The maximum up-field shift for a residual CD_3CD_2HSO line that occurs in the presence of **2** is also smaller (from 2.500 to 2.399 ppm), see Figure S2 [47].

Acetonitrile-d_3. In the context of the above data on the effect of ammonium salt **2** on the residual ^{1}H lines, note a large low-field shift in the CD_2HCN signal (from 1.933 to 2.104 ppm) that was found for a saturated solution of **2** in CD_3CN, see Figure S3 [47]. Pure solvent is relatively durable and can be stored at room temperature, but away from light and moisture [22].

Methanol-d_4. Usually, only the residual line at ~3.3 ppm is used, since the position of the signal from the OH protons (~4.8 ppm) is highly variable; see footnote *a* to Table S2 and Ref. [83]. Its change from 4.837 to 4.588 ppm was found for a saturated solution of salt **2**. At the same time, a down-field shift was observed from 3.305 to ~3.11 ppm for the CD_2HOD signal; see Figure S4 [47].

<u>General comment on Figures S1–S4 [47] (pp. 82–88)</u>

The following stacked 500-MHz ^{1}H NMR titration spectra (only expanded parts shown) were referenced to 'external' TMS (that is, 0.05% *v*/*v* TMS in each of the four NMR solvents used) placed in the internal capillary part of a coaxial tube system. The shifted residual ^{1}H signal of the solvent in titrated solutions is indicated by an asterisk, while the corresponding reference signal from the solvent contained in the capillary insert does not change its position. The added amounts of TBACl (salt **2**) as a titrant are expressed in milligrams.

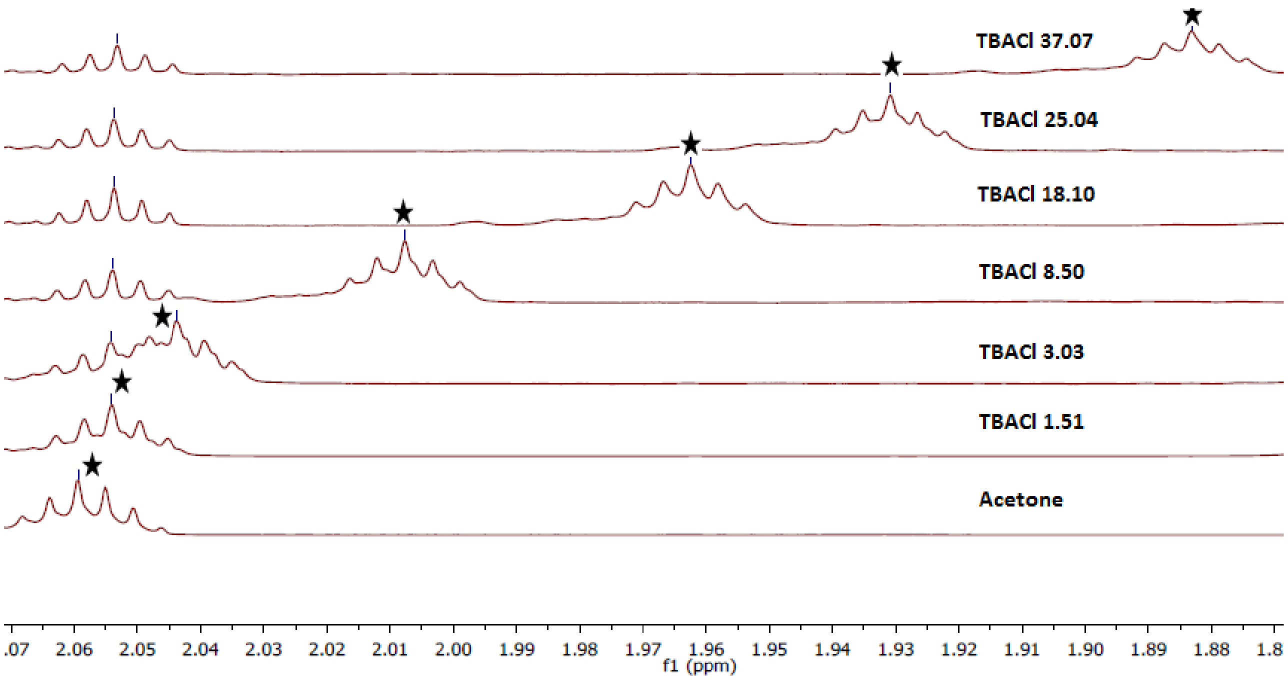

Figure S1. ^{1}H NMR titration plots obtained using salt **2** (TBACl) in acetone-d_6.

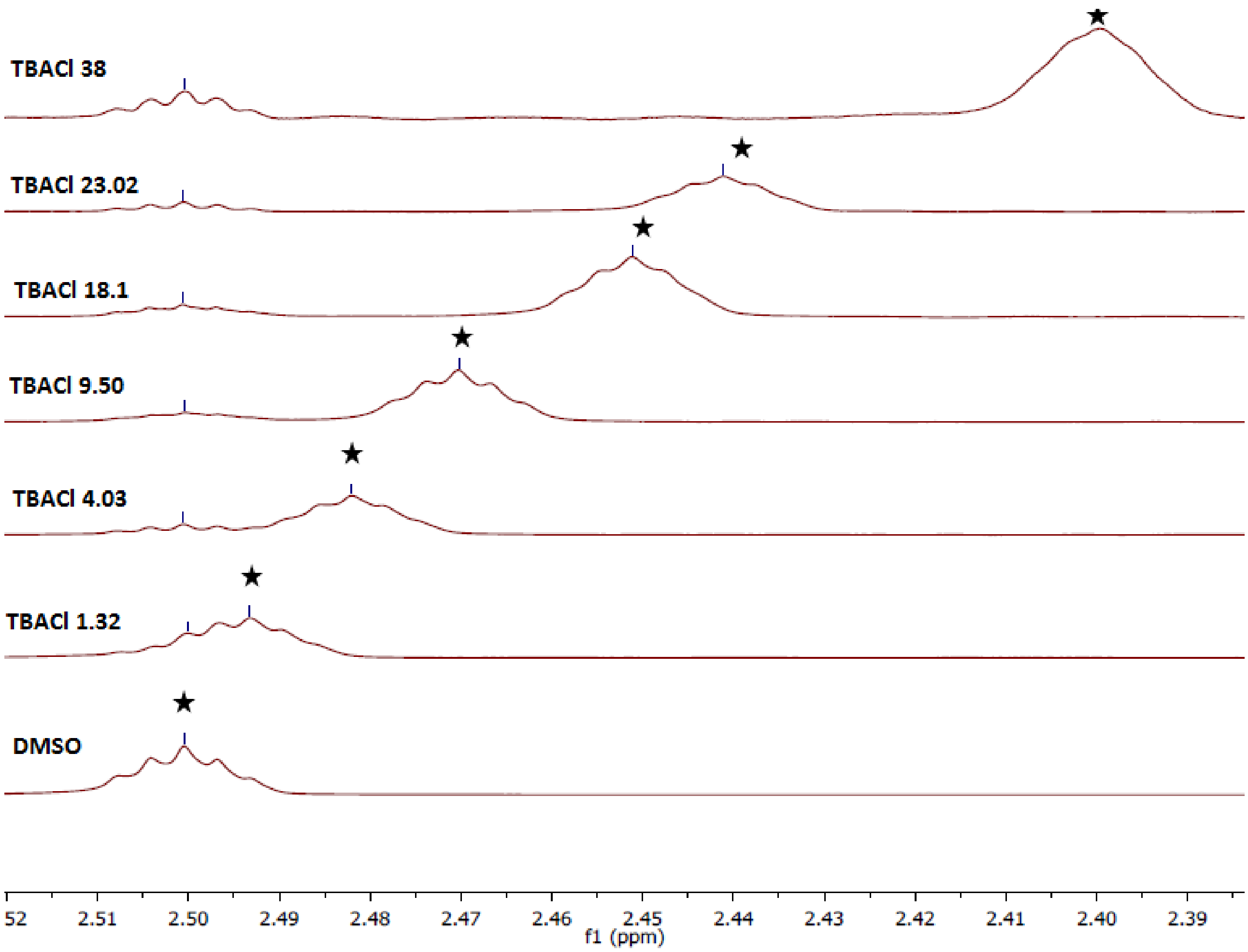

Figure S2. ^{1}H NMR titration plots obtained using salt **2** (TBACl) in DMSO-d_6.

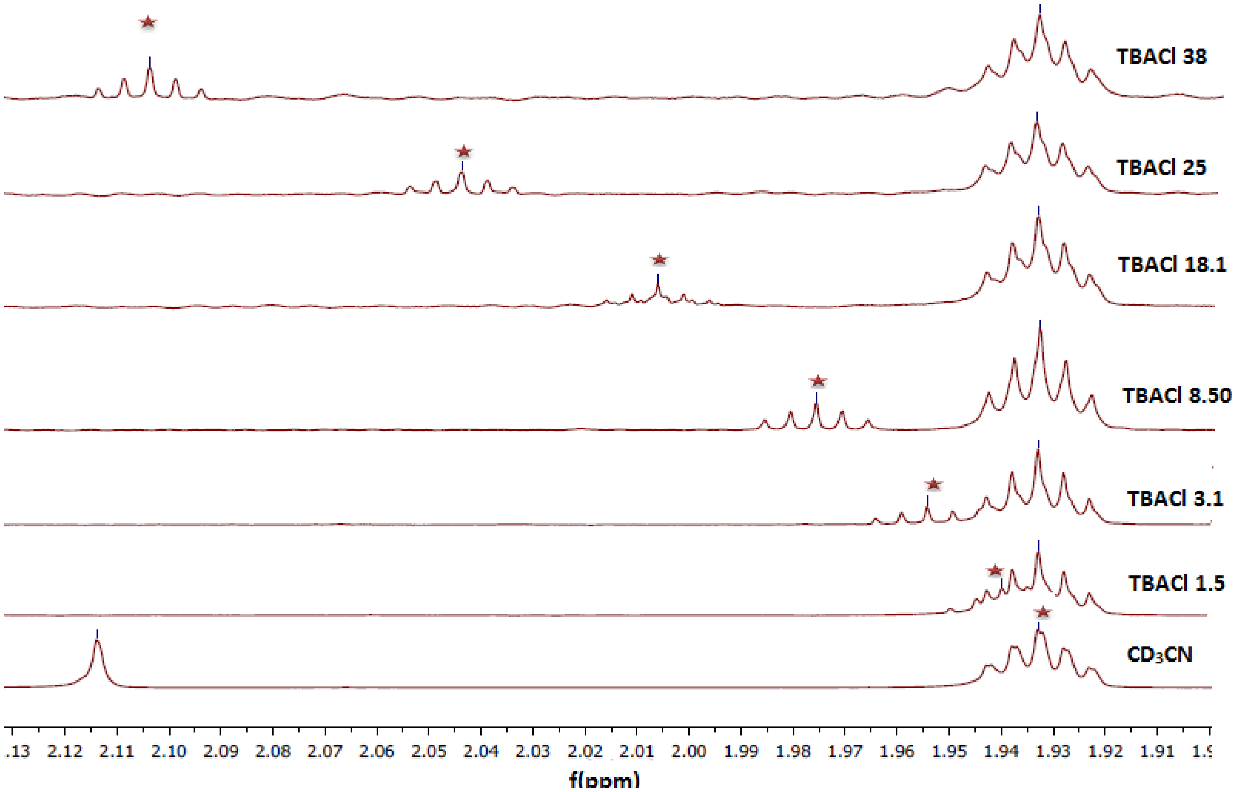

Figure S3. ^{1}H NMR titration plots obtained using salt **2** (TBACl) in acetonitrile-d_3.

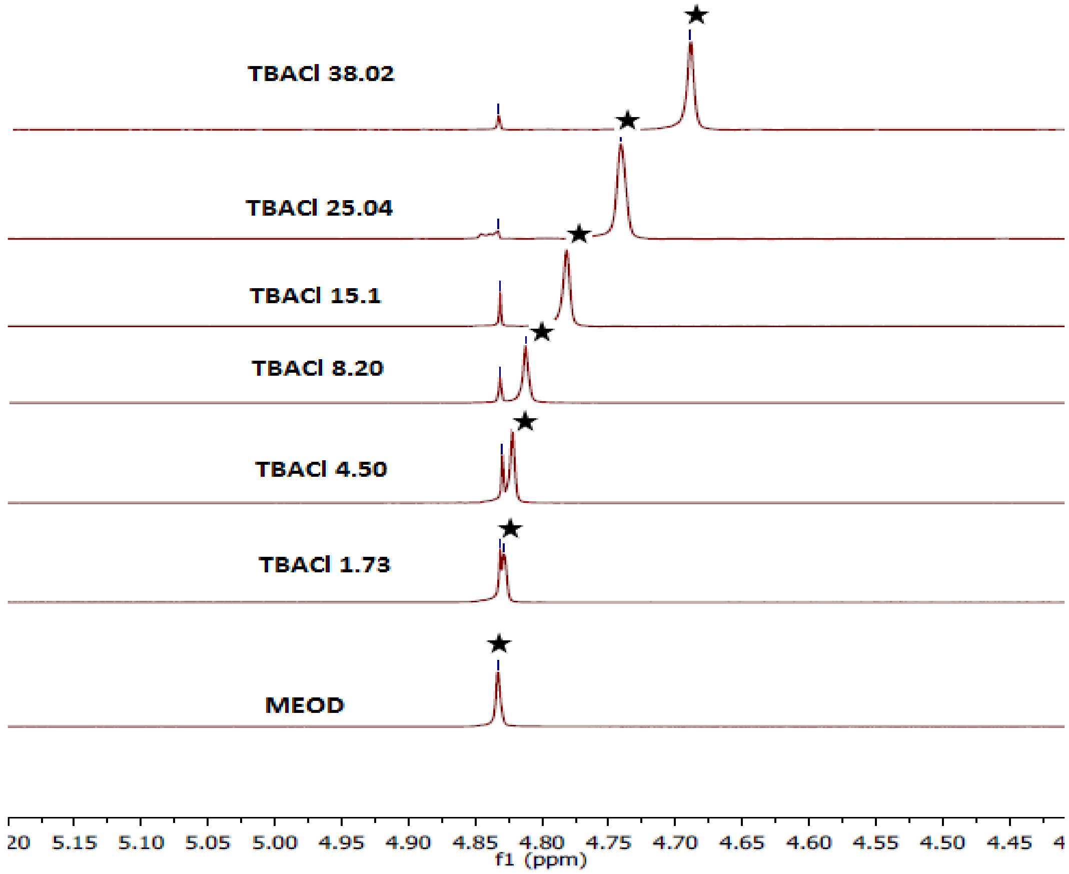

Figure S4. ^{1}H NMR titration plots obtained using salt **2** (TBACl) in methanol-d_4.

Selected cases relating to the ^{13}C spectra

The first reliable values for multiplet ^{13}C signals from deuterated NMR solvents were determined at a frequency of 25.2 MHz in the early 1970s, a time when the NMR community was moving to ^{13}C chemical shifts based on the TMS scale [6,7,51]. All these δ_C data, except slightly underestimated values for acetone-d_6, are close to those currently recommended. However, much better agreement ($\delta_C \leq 0.2$ ppm) is found for the more recent δ_C data set collected at MSD [13]; see the main text.

Of particular note again are the δ_C data found for ternary mixtures containing **1** as analyte [37]. They changed only by a maximum of ± 0.03 ppm from the comparable values for pure TMS-doped solvents [2]. This omits the signal of its carbonyl group $(CD_3)_2$*C*=O at ~206 ppm, which should be used with care as a low-field internal standard due to the known interaction of this group with electron-accepting moieties [84,85]; see also footnote *e* to Table S4. Once again (*vide supra*), the poor quality of the δ_C data given in the encyclopedic book [28,29] is remarkable.

Furthermore, as with ^{1}H spectra, *it is not known which value of δ_C*, more or less close to the recommended data, *will be correct for a given analyzed sample* due to specific solvent–analyte interactions, the influence of concentration, and sample temperature. However, such differences may be much larger, as is well reflected in the ranges between the outsides found for $CDCl_3$ and DMSO-d_6 (0.11 vs 1.91 ppm, and 0.07 vs 1.7 ppm, respectively), see Tables S2 and S4. All these findings are in line with previous results [86]. On the other hand, one may ask how these slightly different δ_X values were found (and adopted in Method A) because TMS was not present in the solution.

Chloroform-d. In Method A, the ^{13}C spectra are calibrated against the middle line of the 1:1:1 triplet of $CDCl_3$ that appears at ~77.0 ppm. The range of δ_C data reported for this line is quite wide, from 76.0 [87] to 77.91 [88] ppm. The latter δ_C value was determined for a concentrated solution of product **9**. In truth, the authors claim that this δ_C was found with respect to internal TMS, but the provided ^{1}H and ^{13}C spectra contradict this.

Table S4. Compilation of δ_C values reported for signals of six common NMR solvents, ppm (vs. internal TMS).

$CDCl_3$	$(CD_3)_2CO$		$(CD_3)_2SO$	C_6D_6	CD_3CN		CD_3OD	Year	References
76.9$_1$[b,c]	29.2$_2$[b,c]	-	39.5$_6$[b,c]	127.9$_6$[b,c]	-	-	-	1972	[6,7]
77.0	28.1	204.4	39.5	-	-	-	47.1	1974	[89]
77.0	29.8	205.7	39.7	128.0	1.2	117.8	49.0	1976-1981	[20,90,91]
77.0	29.8	206.0	39.5	128.0	1.3	118.2	49.0	≤ 1978-2011	[1,13-19]
77.0[b]	29.8	206.5	39.7	128.0[b]	1.3	118.2	49.0	1978-2015	[39,92,93]
77.7[d]	30.5	205.1	39.5	128.7	1.28	118.1	49.0	1988-1992	[35,36,94]
77.75	*29.51$_8$*	*205.93$_3$*	*40.75$_7$*	*127.92$_7$*	-	-	-	1991	[8]
77.23	29.92	206.68	39.51	128.39	1.39	118.69	49.15	≥ 1993	[22,23]
**77.01**	**29.83**	**206.18**	39.57	**128.03**	1.25	118.10	**49.05**	1993	[95]
77.16	**29.84**	**206.26**[e]	**39.52**	**128.06**	**1.32**	118.26	**49.00**	≥ 1997-2010	[24,25]
77.02	29.83	206.09	39.44	-	1.34	118.46	49.06	1999-2015	[27]
76.98[f]	28.92[f]	205.19[f]	39.98[f]	127.68[f]	0.30[f]	117.31[f]	47.84[f,g]	2003-2022	[30,42]
76.9	29.2	204.1	39.6	128.4	1.3	117.7	49.3	2003-2011	[28,29]
**77.01**	**29.83**	206.70	**39.46**	**128.04**	**1.35**	**118.34**	**49.05**	2004	[2]
77	30.7	206.7	[35.2][h]	128	[39.5][h]	118.2	49.0	2005	[31]
77.2	29.9	206.7	39.5	128.4	1.4	118.7	49.2	2009-2016	[33,34]
77.0	29.8	206.0	39.5	128.0	1.3	118.3	49.0	2009-2020	[35,36]
**77.02**	**29.84**	**206.1**	**39.45**	**128.04**	**1.38**	**118.36**	**49.04$_5$**	2010	[37]
77.2	29.9	206.7	39.5	128.4	-	-	-	2016	[40]
77.0	29.9	207.0	39.51	128.4	1.39	118.69	49.15	2017	[96]
77.0	29.8	206.0	39.5	128.0	1.3	118.2	49.0	2022	[41]
**77.02**	**29.80**	**206.07**	**39.41**	**128.00**	**1.38**	**118.37**	**49.04$_5$**	2022-2023	[43,98]
**77.01**	**29.83**	**206.15**	**39.46**	**128.03**	**1.36**	**118.36**	**49.04**		**Recommended data**
[76.0]			(39.1)					1998-2014	For details, see text
[77.91]			[40.80]			[118.77]	[49.86]	2017-2022	For details, see text

[a] Uncertainties below ± 0.07 ppm are not given. 'Absolute' data in italics were obtained for TMS-free solvents by the MAS technique; δ_C = 0.000 ppm was assumed for neat liquid TMS. [b] ± 0.5 ppm. [c] Determined at 38 °C. [d] A printing error [21] corrected [92] to 77.0 ppm. [e] ± 0.13 ppm. [f] Relative to the ^{1}H frequency of TMS in the same solvent × factor (Ξ/100), that for the ^{13}C nucleus is 0.25145020 [97]. To obtain usually observed δ_H values, some correction factors (terms) must be added to these raw calculation data (see main text). [g] The new [98] value δ_C = 47.58$_5$ ppm. [h] Obviously, incorrect values. [g] Ref. [98].

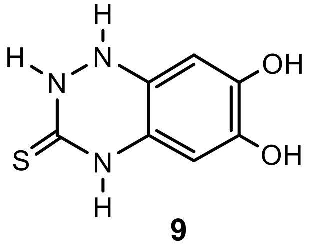

9

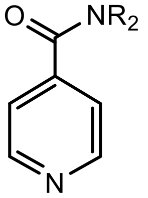

10a R = Me
10b R = Et
10c R = *i*Pr .

Analytes of a similar type can be thought to exert comparable CISs of the signals of the solvent medium used. In fact, in the $^{13}C\{^1H\}$ spectra of three homologous amides **10a**–**c** dissolved in TMS-doped $CDCl_3$, the solvent signal appeared at 77.17, 77.21, and 77.07 ppm for **10a**, **10b**, and **10c**, respectively [99]. Closer inspection of the associated ^1H NMR spectra recorded for these samples allows estimation of the relative analyte/solvent ratio as ~3, ~6, and 1, respectively. The last sample was the most dilute solution, for which the δ_C value is closest to the 'reference' data for pure $CDCl_3$ with TMS, as expected.

Other NMR solvents. The example of acetone-d_6 is discussed above. It is difficult to comment on the three outlier δ_C values (40.80 [100], 118.77 [101], and 49.86 [101] ppm) reported for DMSO-d_6, CD_3CN, and CD_3OD, respectively. It should be noted that the same authors adopted greatly overestimated δ_C values for CD_3CN and especially for CD_3OD. It is difficult to find reasons for this situation. In turn, the solvent signal of DMSO-d_6 at 39.1 ppm was adopted in an interlaboratory study involving 14 participants [102].

Table S5. Compilation of δ_D values reported for ^2H signals of six common NMR solvents used as the reference standards, ppm

$CDCl_3$	$(CD_3)_2CO$	$(CD_3)_2SO$	C_6D_6	CD_3CN	CD_3OD	$CD_3O\mathit{D}$	Year	References
7.229	2.031	2.493[a]	7.185	1.913	3.294	-	2004[b]	[2]
7.24	2.04	2.49	7.16	1.93	3.30	-	2006-2022	[32,103,104]
7.290	2.097	2.559	-	1.974	3.350	4.878	2022[c]	[42]
7.290	2.101	2.560	-	1.974	3.354	5.077	2022[c]	[43]
7.290	1.934_5	2.620		1.960	3.245	4.968	2022[d,e]	[43]

[a] The δ_D value refers to acetone-d_6 (2.051 ppm). [b] Found at 27 °C *vs*. ^2H signal (in natural abundance) of dissolved TMS (3 wt/vol %). [c] Found at 25 °C relative to the ^1H signal of dissolved TMS. [d] Found relative to the ^1H signal of dilute TMS in $CDCl_3$ at 25 °C. [e] Found at 25 °C relative to the ^1H signal of TMS in the same solvent.

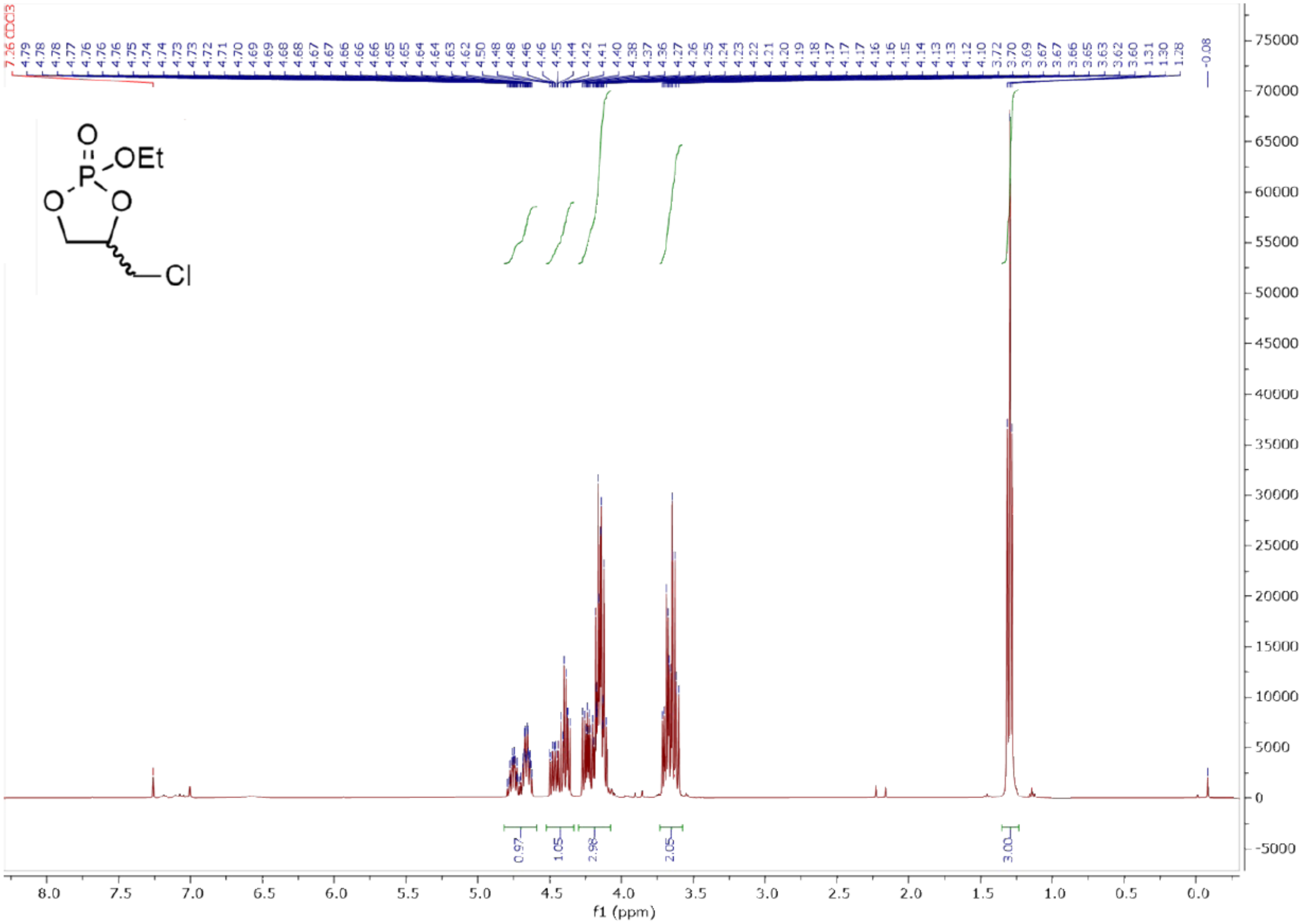

Figure S5. 400 MHz ^{1}H NMR spectrum of 4-(chloromethyl)-2-ethoxy-1,3,2-dioxaphospholane 2-oxide (compound **1** in the main text) recorded in $CDCl_3$ solution (mixture of stereoisomers) [105] (Electronic Supplementary Information, p. S46). The second small signal at about δ_H 0.07 ppm is attributable to silicone grease [24]. Reproduced with permission from the Royal Society of Chemistry.

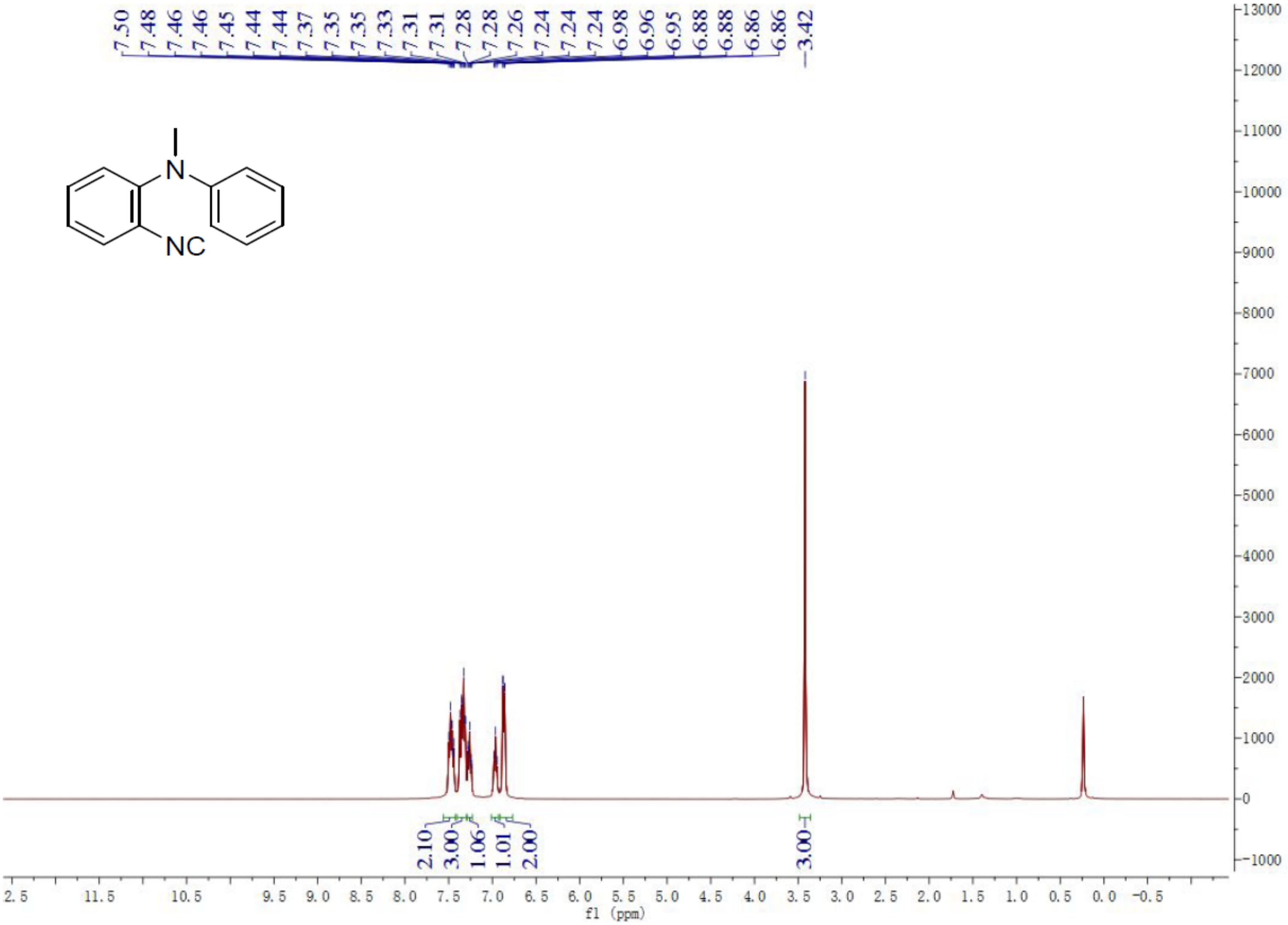

Figure S6. 400 MHz ^{1}H NMR spectrum 2-isocyano-*N*-methyl-*N*-phenylaniline (compound **3** in the main text) recorded in $CDCl_3$ solution [44] (Supporting Information, p. S14). Adapted with permission from *J. Org. Chem.* **2022,** *87*, 16542−16549. Copyright 2022 American Chemical Society.

Computational results obtained in this work

Initial models of the 1:1 hydrogen-bonded intermolecular complexes **7** [60] and **8** [61] were obtained for the gas phase at the HF/6-31G level by applying the HyperChem program [106]. (It is worth noting that the CH⋯π T-shape interaction [60] in system **6** is an example of 'unconventional hydrogen bonds' because the aromatic ring acts as an H acceptor [107].) More advanced calculations were carried out with the Gaussian 16 package [108], using the PCMODEL 8.5 program [109] as an interface. Finally, the molecular structures of both the complexes studied and two internal reference markers (TMS, C*H*Cl_3) were optimized at the standard [63,64] B3LYP/6-311+G(d,p) level. The free (isolated) species **7** and **8** thus optimized, with the Cartesian coordinates of their atoms given in Table S7, were subjected to further single-point GIAO-based [110] predictions at a typical [62,64–66] mPW1PW91/6-311+G(2d,p) level. Two versions of these DFT-NMR calculations were performed: (*a*) for isolated species in the gas phase and (*b*) for the $CHCl_3$ solution simulated by the IEF-PCM scheme [111]. The predicted magnetic shielding values (σ_Hs) were then standardly converted to δ_H values using TMS of the T_d symmetry [112] as a reference standard; $\delta_{H,i} = \sigma_{H,TMS} - \sigma_{H,i}$, where *i* = proton in the system under analysis [113]. All results obtained are summarized in Table S6. The fully optimized molecular structures of both complexes are shown in Figure S4, using graphical representations created with ChemCraft [114].

Table S6. The δ_H data for species **7** and **8** as well as for TMS and C*H*Cl_3 as internal reference standards predicted *via* GIAO-DFT-NMR calculations

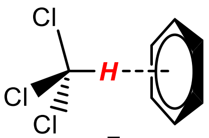

7

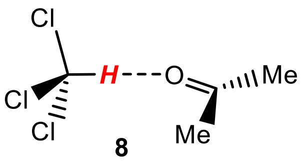

8

Row	Quantity[a]	TMS[b]	C*H*Cl_3[c]	Complex **7**[c]	Complex **8**
			**Gas phase**		
1	E_{total}, au	-449.27205	-1419.37966	-1651.69403	-1612.60510
2	*G*, au	-449.15514	-1419.38807	-1651.61852	-1612.54693
3	f.v.f., cm^{-1}	143.77	261.40	6.68	6.14
4	σ_H, ppm	31.7814	24.0854	26.6371[d]	22.1170[d]
5	σ_C, ppm	187.0296			
6	σ_{Si}, ppm	342.9505			
7	δ_H, ppm	0.0000	7.6960	5.1443[d]	9.6644[d]
			**Simulated solutions (IEF-PCM scheme)**		
8	σ_H, ppm	31.776_6	23.951_8	26.632_0[d]	22.091_7[d]
9	δ_H, ppm	0.0000	***7.824_8***	***5.144_6***[d]	***9.684_9***[d]
			**The C*H*Cl_3 line in real solvents ($CDCl_3$, C_6D_6 and acetone-*d_6*)** [24,25]		
10	δ_H, ppm	0.00	***7.26***	***6.15***	***8.02***

[a] Abbreviations used: E_{total} = total electronic energy, *G* = Gibbs free energy, f.v.f. = first vibrational frequency. [b] T_d-symmetric system. [c] C_{3v}-symmetric system. [d] For the C*H*Cl_3 part of the complex.

Analysis of the theoretical values given in rows 7 or 9 of Table S6 for both systems analyzed indicates that in the presence of benzene or acetone in the $CDCl_3$ solution, they predict a significant shift of the residual C*H*Cl_3 signal to a lower and higher frequency, respectively, which is consistent with experimental observation (row 10). Furthermore, it can be mentioned that the σ_H and σ_C data calculated for TMS in the gas phase (rows 4 and 5) agree well with the data found for its isolated molecules (30.785 and 188.04 ppm, respectively) [115]. However, for the ^{29}Si nuclei (row 6), the agreement of theory with the measurement (σ_{Si} = 376.4 ppm) [115] is much worse, strongly suggesting that the approximations used in both steps of the current NMR calculations are not suitable for σ_{Si} predictions. The importance of including core-valence basis sets in the accurate calculation of nuclear shieldings for third-row elements has recently been demonstrated by Rzepiela *et al*. [116]. In turn, it should be noted that there is a very small difference in the δ_H values calculated for the C*H*Cl_3 line from the $CHCl_3$ part of species **7** and **8**, compared to free C*H*Cl_3, when going from the gas state to the solution state.

Table S7. Optimized geometries of complexes **7** and **8** in Cartesian coordinates, in Angströms, obtained at the B3LYP/6-311+G(*d*,*p*) level

System **7**	System **8**
C,0,1.2744962212,-1.3339520758,1.7408499219	C,0,1.5634057195,1.498513074,-0.2129262114
C,0,1.8384960686,-1.2736981959,0.4659625929	C,0,1.5032906759,-0.0133869549,-0.1785768556
C,0,2.1618696659,-0.0381881963,-0.0961838447	C,0,2.8240333631,-0.7523400782,-0.171069901
C,0,1.9202628766,1.1371165309,0.6161005207	O,0,0.4457715842,-0.6122154776,-0.1572270181
C,0,1.3562621463,1.0768366217,1.8909862288	H,0,2.2516505253,1.8426028472,-0.9903711187
C,0,1.0328798509,-0.1586613389,2.4531541217	H,0,1.9538022257,1.8654264112,0.742622902
H,0,1.0216969477,-2.2937735508,2.1769312526	H,0,0.5714139883,1.9166562867,-0.3782087556
H,0,2.0246371851,-2.1868077049,-0.0881903327	H,0,3.4983590749,-0.3379694763,0.5840082915
H,0,2.5985130664,0.0087309734,-1.087381502	H,0,3.3158199792,-0.6246169369,-1.1415249912
H,0,2.1699365102,2.0972014365,0.1786042278	H,0,2.6607258805,-1.8133466422,0.0118016645
H,0,1.1669907127,1.9900716551,2.4437156037	H,0,-1.6420399464,-0.3300961432,-0.1597350077
H,0,0.5930600494,-0.2053912007,3.4430419022	C,0,-2.6894364887,-0.0493542534,-0.1635125109
Cl,0,-2.7020964641,-1.3667379636,0.1471182462	Cl,0,-3.3741309009,-0.4378029256,1.4415200073
C,0,-1.8099582239,0.1104666562,-0.3200820956	Cl,0,-3.5106730424,-0.9684213146,-1.4572486821
Cl,0,-2.6023156498,1.5752022006,0.330332989	Cl,0,-2.7664256382,1.7151965837,-0.4824358133
H,0,-0.8203171788,0.0497954446,0.1151660926	
Cl,0,-1.6192145091,0.2145121079,-2.0946557535	

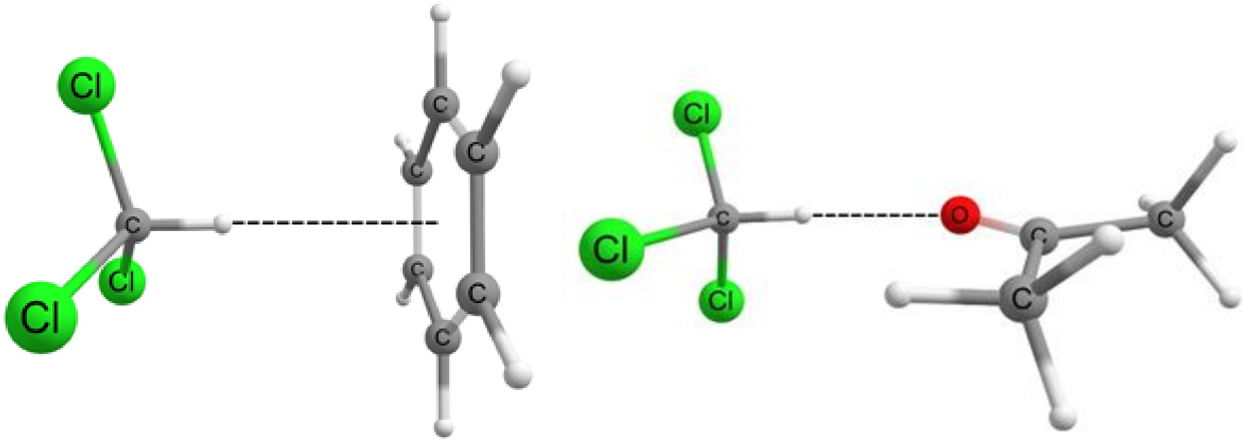

Figure S7. The 3D shapes of complexes **7** (left) and **8** (right) generated with ChemCraft [114].

References for Supplementary Materials

(1) Kegley, S. E.; Pinhas, A. R. *Problems and Solutions in Organometallic Chemistry*; University Science Books: Mill Valley, 1986, p. 9.

(2) Hatada, K.; Kitayama, T. *NMR Spectroscopy of Polymers*; Springer-Verlag: Berlin, 2004, pp. 1−42 and refs cited therein.

(3) Hoffman, R. E.; Becker, E. D. Temperature dependence of the ^{1}H chemical shift of tetramethylsilane in chloroform, methanol, and dimethylsulfoxide. *J. Magn. Reson.* **2005**, *176*, 87−98.

(4) Guzman, A. L.; Hoye, T. R. TMS is Superior to Residual C*H*Cl_3 for Use as the Internal Reference for Routine ^{1}H NMR Spectra Recorded in $CDCl_3$. *J. Org. Chem*. **2022**, *87*, 905−909 and refs therein.

(5) Hoffman, R. Magnetic susceptibility measurement by NMR: 2. The magnetic susceptibility of NMR solvents and their chemical shifts. *J. Magn. Reson*. **2022**, *335*, 107105.

(6) Levy, G. C.; Nelson, G. L. *Carbon-13 Nuclear Magnetic Resonance for Organic Chemists*; Wiley-Interscience: New York, 1972, p. 23.

(7) Levy, G. C.; Cargioli, J. D. Carbon-13 Chemical Shifts on the TMS Scale. *J. Magn. Reson*. **1972**, *6*, 143–144.

(8) Hayashi, S.; Yanagisawa, M.; Hayamizu, K. Nuclear Magnetic Resonance Chemical Shifts of Pure Organic-Solvents Determined by Magic Angle Spinning. *Anal. Sci.* **1991**, *7*, 955−957.

(9) Jackowski, K.; Jaszuński, M.; Wilczek, M. Alternative Approach to the Standardization of NMR Spectra. Direct Measurement of Nuclear Magnetic Shielding in Molecules. *J. Phys. Chem. A* **2010**, *114*, 2471−2475.

(10) Garbacz, P.; Jackowski, K. Referencing of ^{1}H and ^{13}C NMR shielding measurements. *Chem. Phys. Lett.* **2019**, *728*, 148−152.

(11) Silverstein, R. M.; Bassler, G. C. *Spectrometric Identification of Organic Compounds*, 2nd ed.; John Wiley & Sons, Inc.: New York, 1967, p. 135.

(12) Gordon, A. J.; Ford, R. A. *The Chemist's Companion – A Handbook of Practical Data, Techniques, and References*; John Wiley & Sons: New York, 1972, pp. 249−251.

(13) *Deuterated NMR Solvents–Handy Reference Data*; Merck Sharp & Dohme Canada Ltd., Isotope Division, Pointe Claire-Dorval, Quebec, Canada.

(14) *Deuterated NMR Solvents–Handy Reference Data.* https://scs.illinois.edu/system/files/inline-files/Deuterated_Solvents.pdf (accessed on 21 May 2023).

(15) *Deuterated NMR Solvent Table.* https://web.stanford.edu/group/chem-NMR/help_docs/nmr_solvents.htm (accessed on 21 May 2023).

(16) Kalinowski, H.-O.; Berger, S.; Braun, S. *^{13}C-NMR-Spektroskopie*; Georg Thieme Verlag: Stuttgart, 1984, pp. 73−74.

(17) Kalinowski, H.-O.; Berger, S.; Braun, S. *Carbon-13C NMR Spectroscopy*; John Wiley & Sons: Chichester, 1988, pp 85−86.

(18) *Spectroscopy Catalogue*. Sigma-Aldrich Co., 1997, p. 37.

(19) Reichardt, C.; Welton, T. *Solvents and Solvent Effects in Organic Chemistry*, 4th ed.; Wiley-VCH Verlag GmbH & Co. KGaA: Weinheim, 2011, pp. 564−567 and refs cited therein.

(20) Reichardt, C. *Solvents and Solvent Effects in Organic Chemistry*, 1st ed.; Verlag Chemie GmbH: Weinheim, 1979, pp. 277−278 and refs cited therein.

(21) *Handbuch der Instrumentellen Analytik. NMR-Spektroskopie*; E. Merck: Darmstadt, 1988.

(22) *CIL's NMR Solvent Data Chart*. https://www.isotope.com/ (accessed on 21 May 2023).

(23) Zerbe, O.; Jurt, S. *Applied NMR Spectroscopy for Chemists and Life Scientists*; Wiley-VCH Verlag GmbH& Co. KGaA: Weinheim, 2014. p. 28. Note that the authors erroneously cited another book as the source of the reference data δ_X instead of Ref. [22]. For a full explanation of this issue, see the main text of the paper.

(24) Gottlieb, H. E.; Kotlyar, V.; Nudelman, A. NMR Chemical Shifts of Common Laboratory Solvents as Trace Impurities. *J. Org. Chem.* **1997**, *62*, 7512−7515.

(25) Fulmer, G. R.; Miller, A. J. M.; Sherden, N. H.; Gottlieb, H. E.; Nudelman, A.; Stoltz, B. M.; Bercaw, J. E.; Goldberg, K. I. NMR Chemical Shifts of Trace Impurities: Common Laboratory Solvents, Organics, and Gases in Deuterated Solvents Relevant to the Organometallic Chemist. *Organometallics* **2010**, *29*, 2176−2179.

(26) Babij, N. R.; McCusker, E. O.; Whiteker, G. T.; Canturk, B.; Choy, N.; Creemer, L. C.; De Amicis, C. V.; Hewlett, N. M.; Johnson, P. L.; Knobelsdorf, J. A.; Li, F.; Lorsbach, B. A.; Nugent, B. M.; Ryan, S. J.; Smith, M. R.; Yang, Q. NMR Chemical Shifts of Trace Impurities: Industrially Preferred Solvents Used in Process and Green Chemistry. *Org. Process Res. Dev*. **2016**, *20*, 661−667.

(27) *Spectral Database for Organic Compounds.* National Institute of Advanced Industrial Science and Technology, Japan. https://sdbs.db.aist.go.jp/sdbs/cgi-bin/direct_frame_top.cgi (accessed on 21 May 2023).

(28) Bruno, T. J.; Svoronos, P. D. N. *CRC Handbook of Basic Tables for Chemical Analysis*. 2nd ed.; CRC Press: Boca Raton, 2003 and refs cited therein.

(29) Bruno, T. J.; Svoronos, P. D. N. *CRC Handbook of Basic Tables for Chemical Analysis*. 3rd ed.; CRC Press: Boca Raton, 2011 pp 484−486 and refs cited therein.

(30) Hoffman, R. E. Variations on the chemical shift of TMS. *J. Magn. Reson*. **2003**, *163*, 325−331.
(31) Balci, M. *Basic ^{1}H- and ^{13}C-NMR Spectroscopy*; Elsevier: London, 2005, p. 35.
(32) Hoffman, R. E. Standardization of chemical shifts of TMS and solvent signals in NMR solvents. *Magn. Reson. Chem*. **2006**, 44, 606−616.
(33) Claridge, T. D. W. *High-Resolution NMR Techniques in Organic Chemistry*. 2nd ed.; Elsevier Ltd.: Amsterdam, 2009, p. 60.
(34) Claridge, T. D. W. *High-Resolution NMR Techniques in Organic Chemistry*. 3rd ed.; Elsevier Ltd.: Amsterdam, 2016, p. 90.
(35) Pretsch, E.; Bühlmann, P; Badertscher, M. *Structure Determination of Organic Compounds. Tables of Spectral Data*. 4td ed.; Springer-Verlag: Berlin, 2009, pp. 153−154, 239−240.
(36) Pretsch, E.; Bühlmann, P; Badertscher, M. *Structure Determination of Organic Compounds. Tables of Spectral Data*. 5td ed.; Springer-Verlag GmbH Germany: Berlin, 2020, pp. 160−162, 251−252.
(37) Chalmers, B. A.; Chen, A. P.-J.; Savage, G. P.; Williams, C. M. Cubane: A New NMR Internal Standard. *Aust. J. Chem*. **2010**, *63*, 1108−1110.
(38) Richards, S. A.; Hollerton, J. C. *Essential Practical NMR for Organic Chemistry*; John Wiley & Sons: Chichester, 2011, p. 16.
(39) Silverstein, R. M.; Webster, F. X.; Kiemle, D. J.; Bryce, D. L. *Spectrometric Identification of Organic Compounds*, 8th ed.; John Wiley & Sons, Inc.: New York, 2015, pp. 187 and 225.
(40) Metz, K. R. Nuclear Magnetic Resonance (NMR) Spectroscopy. In *Handbook of Measurement in Science and Engineering*; Kutz, M., Ed.; John Wiley & Sons, Inc.: Hoboken, 2016; Vol. 3, p. 2547.
(41) *NMR Deuterated Solvent Properties Reference Chart*. https://www.sigmaaldrich.com/PL/pl/technical-documents/technical-article/analytical-chemistry/nuclear-magnetic-resonance/nmr-deuterated-solvent-properties-reference (accessed on 21 May 2023).
(42) *Chemical shift referencing*. http://chem.ch.huji.ac.il/nmr/whatisnmr/chemshift.html (accessed on 21 May 2023).
(43) *NMR Solvent reference shift. Version 4.2.* http://chem.ch.huji.ac.il/nmr/software/solvent.html (accessed on 21 May 2023).
(44) Yuan, S.; Liu, X.; Huang, Z.; Gui, S.; Diao, Y.; Peng, Y.-Y.; Ding, Q. Tetrabutylammonium Chloride-Induced Cascade Radical Addition/Cyclization of *O*-Isocyanodiaryl Amines: A Novel Protocol for the Synthesis of 11-Trifluoromethylated Dibenzodiazepines. *J. Org. Chem.* **2022**, *87*, 16542−16549.
(45) Sleem, H. F. *Synthesis of New Macrocyclic Polyamides and Polysulfonamides and a Study of their Complexation Behavior using ^{1}H-NMR and Mass Spectrometry.* Ph.D. Thesis, Memorial University of Newfoundland, St. John's, Canada, 2013, pp. 144−159.
(46) Sleem, H. F.; Dawe, L. N.; Rahman, S.; Georghiou, P. E. Halide ion effect on the chloroform chemical shift in supramolecular complexation studies with tetra-*n*-butylammonium salts: a ^{1}H NMR and X-ray study. *Supramol. Chem.* **2014**, *26*, 579−582.
(47) Assiri, Y. *Complexation Properties of Upper- and Lower-rim Functionalized Calix[4]arenes*. M.Sc. Thesis, Memorial University of Newfoundland, St. John's, Canada, 2014, Chapter 3, pp. 76−95.
(48) Assiri, Y.; Rahman, S.; Georghiou, P. E. Halide ion effect on the ^{1}H NMR chemical shifts of the residual protons in commonly employed deuterated solvents with tetra-*n*-butylammonium chloride − Part 2. *Supramol. Chem*. **2016**, *28*, 6−9.
(49) Georghiou, P. E. (Memorial University of Newfoundland, St. John's, Canada). Personal communication, 2022. The FID files of ^{1}H NMR spectra taken for TBACl are available from the author upon request.
(50) Laszlo, P.; Speert, A.; Ottinger, R.; Reisse, J. Reconsideration of the Internal Tetramethylsilane Reference for Proton Magnetic Resonance Studies. *J. Chem. Phys.* **1968**, *48*, 1732−1735.
(51) Bacon, R.; Maciel, G. E. Solvent Effects on the Five Shielding Constants In Tetramethylsilane and Cyclohexane. *J. Am. Chem. Soc.* **1973**, *95*, 2413−2426.
(52) Jutila, M. Influence of an Internal Reference on ^{1}H NMR Solvent Shifts. Determination of Reference Independent ASIS Values. *Acta Chem. Scand.* **1981**, *B35*, 503−506 and refs cited therein.
(53) Pauli, G. F.; Chen, S.-N.; Lankin, D. C.; Bisson, J.; Case, R. J. *et al*. Essential Parameters for Structural Analysis and Dereplication by ^{1}H NMR Spectroscopy. *J. Nat. Prod*. **2014**, *77*, 1473−1487.
(54) Pauli G. F.; Niemitz, M.; Bisson, J.; Lodewyk, M. W.; Soldi, C. *et al.* Toward Structural Correctness: Aquatolide and the Importance of 1D Proton NMR FID Archiving. *J. Org. Chem*. **2016**, *81*, 878−889.
(55) Desando, M. A.; Lahajnar, G.; Plavec, J. Molecular Interactions and Mechanisms in the ^{1}H NMR Relaxation of Residual $CHCl_3$ in Deuterochloroform Solution of a Two-Chain Ionic Surfactant. *J. Solution Chem*. **2018**, *47*, 1246−1268.
(56) Jones, I. C.; Sharman, G. J.; Pidgeon, J. Spectral Assignments and Reference Data ^{1}H and ^{13}C NMR data to aid the identification and quantification of residual solvents by NMR spectroscopy. *Magn. Reson. Chem*. **2005**, *43*, 497−509.

(57) Wu, Z.; Jäger, M.; Buter, J.; Minnaard, A. J. A protecting group-free synthesis of the Colorado potato beetle pheromone. *Beilstein J. Org. Chem*. **2013**, *9*, 2374−2377.
(58) Chan, D.; Cronin, L.; Duckett, S. B.; Hupfield, P.; Perutz, R. N. Synthesis, structure and reactivity of *N*,*O*-metallacyclic (dicarbonyldiazene) platinum complexes. *New J. Chem*. **1998**, *22*, 511−516.
(59) Popova, T.; Dymova, M. A.; Koroleva, L. S.; Zakharova, O. D.; Lisitskiy, V. A.; Raskolupova, V. I.; Sycheva, T.; Taskaev, S.; Silnikov, V. N.; Godovikova, T. S. Homocystamide Conjugates of Human Serum Albumin as a Platform to Prepare Bimodal Multidrug Delivery Systems for Boron Neutron Capture Therapy. *Molecules* **2021**, *26*, 6537.
(60) Tsuzuki, S.; Honda, K.; Uchimaru, T.; Mikami, M.; Tanabe, K. The Interaction of Benzene with Chloro- and Fluoromethanes: Effects of Halogenation on CH/π Interaction. *J. Phys. Chem. A* **2002**, *106*, 4423−4428 and refs cited therein.
(61) Golubev, V. A.; Gurina, D. L.; Kumeev, R. S. Self-Diffusion and Heteroassociation in an Acetone−Chloroform Mixture at 298 K. *Russ. J. Phys. Chem. A*, **2018**, *92*, 75−78.
(62) Nazarski, R. B.; Wałejko, P.; Witkowski, S. Multi-conformer molecules in solutions: an NMR-based DFT/MP2 conformational study of two glucopyranosides of a vitamin E model compound. *Org. Biomol. Chem.* **2016**, *14*, 3142−3158.
(63) Adamson, J.; Nazarski, R. B.; Jarvet, J.; Pehk, T.; Aav, R. Shortfall of B3LYP in Reproducing NMR J_{CH} Couplings in Some Isomeric Epoxy Structures with Strong Stereoelectronic Effects: A Benchmark Study on DFT Functionals. *ChemPhysChem* **2018**, *19*, 631−642.
(64) Ciechańska, M.; Jóźwiak, A.; Nazarski, R. B.; Skorupska, E. A. Unexpected Rearrangement of Dilithiated Isoindoline-1,3-diols into 3-Aminoindan-1-ones via *N*-Lithioaminoarylcarbenes: A Combined Synthetic and Computational Study. *J. Org. Chem.* **2019**, *84*, 11425−11440.
(65) Hägele, G.; Nazarski, R. B.; Schmitz, A.; Xing, S.; Janiak, C. ^{1}H NMR spectra, structure, and conformational exchange of S-*n*-alkyltetrahydrothiophenium cations of some ionic liquids. *Phosphorus Sulfur Silicon Relat. Elem.* **2022**, *197*, 788−798.
(66) Cloutier, M.; Lavoie, S.; Gauthier, C. C7 Epimerization of Benzylidene-Protected β-D-Idopyranosides Brings Structural Insights into Idose Conformational Flexibility. *J. Org. Chem.* **2022**, *87*, 12932−12953.
(67) Muhamadejev, R.; Melngaile, R.; Paegle, P.; Zibarte, I.; Petrova, M.; Jaudzems, K.; Veliks, J. Residual Solvent Signal of $CDCl_3$ as a qNMR Internal Standard for Application in Organic Chemistry Laboratory. *J. Org. Chem.* **2021**, *86*, 3890−3896.
(68) *Deuterated Chloroform*. https://www.ckgas.com/wp-content/uploads/2015/04/deuterated_chloroform.pdf (accessed on 21 May 2023).
(69) Kawai, S. Discussion on Decomposition of Chloroform. *J. Pharm. Soc. Jpn.* **1966**, *86*, 1125−1132.
(70) Alapi, T.; Dombi, A. Direct VUV photolysis of chlorinated methanes and their mixtures in an oxygen stream using an ozone producing low-pressure mercury vapour lamp. *Chemosphere* **2007**, *67*, 693−701.
(71) Teipel, J.; Gottstein, V.; Hölzle, E.; Kaltenbach, K.; Lachenmeier, D. W.; Kuballa, T. An Easy and Reliable Method for the Mitigation of Deuterated Chloroform Decomposition to Stabilise Susceptible NMR Samples. *Chemistry* **2022**, *4*, 776−785 and refs cited therein
(72) Hungerford, N. L.; McKinney, A. R.; Stenhouse, A. M.; McLeod, M. D. Selective manipulation of steroid hydroxyl groups with boronate esters: efficient access to antigenic C-3 linked steroid−protein conjugates and steroid sulfate standards for drug detection. *Org. Biomol. Chem*. **2006**, 4, 3951−3959.
(73) Miles, W. H.; Duca, D. G.; Selfridge, B. R.; Palha De Sousa, C. A.; Hamman, K. B.; Goodzeit, E. O.; Freedman, J. T. Amine-catalyzed epimerization of γ-hydroxybutenolides. *Tetrahedron Lett.* **2007**, *48*, 7809−7812.
(74) Dias, D. A.; Urban, S. Phytochemical Investigation of the Australian Lichens *Ramalina glaucescens* and *Xanthoria parietina*. *Nat. Prod. Commun.* **2009**, *4*, 959−964.
(75) Dialer, L. O.; Selivanova, S. V.; Müller, C. J.; Müller, A.; Stellfeld, T.; Graham, K.; Dinkelborg, L. M.; Krämer, S. D.; Schibli, R.; Reiher, M.; Ametamey, S. M. Studies toward the Development of New Silicon-Containing Building Blocks for the Direct ^{18}F-Labeling of Peptides. *J. Med. Chem*. **2013**, *56*, 7552−7563.
(76) Inayoshi, T.; Hirata, K.; Watanabe, T.; Yamazaki, M. Syntheses and properties of a new series of tetrathiafulvalene derivatives incorporating fused ethyleneoxymethylene and ethylenethiomethylene units, and their charge–transfer complexes. *Synth. Met.* **2015**, *205*, 162−177 and refs cited therein.
(77) Sedrik, R.; Bonjour, O.; Laanesoo, S.; Liblikas, I.; Pehk, T.; Jannasch, P.; Vares, L. Chemically Recyclable Poly(β-thioether ester)s Based on Rigid Spirocyclic Ketal Diols Derived from Citric Acid. *Biomacromolecules* **2022**, *23*, 2685−2696.
(78) Gunda, P.; Russon, L. M.; Lakshman, M. K. Pd-Catalyzed Amination of Nucleoside Arylsulfonates to yield N^6-Aryl-2,6-Diaminopurine Nucleosides. *Angew. Chem. Int. Ed*. **2004**, *43*, 6372−6377.
(79) Salih, A. M.; Ahmad, M. B.; Ibrahim, N. A.; Dahlan, K. Z. H. M.; Tajau, R.; Mahmood, M. H.; Yunus, W. M. Z. W. Synthesis of Radiation Curable Palm Oil−Based Epoxy Acrylate: NMR and FTIR Spectroscopic Investigations. *Molecules* **2015**, *20*, 14191−14211.

(80) Nguyen, T. V. T.; Wodrich, M. D.; Waser, J. Substrate-controlled C–H or C–C alkynylation of cyclopropanes: generation of aryl radical cations by direct light activation of hypervalent iodine reagents. *Chem. Sci.* **2022**, *13*, 12831−12839.
(81) Zhong, T.; Wolcott, M. P.; Liu, H.; Wang, J. Propionylation-modified chitin with improved solubility in green ethanol/water binary solvents for sustainable film and coating applications. *J. Clean. Prod.* **2020**, *250*, 119458.
(82) Mascitti, A.; Lupacchini, M.; Guerra, R.; Taydakov, I.; Tonucci, L.; d'Alessandro, N.; Lamaty, F.; Martinez, J.; Colacino, E. Poly(ethylene glycol)s as grinding additives in the mechanochemical preparation of highly functionalized 3,5-disubstituted hydantoins. *Beilstein J. Org. Chem.* ***2017***, *13*, 19−25.
(83) It is generally accepted that this is the common signal of CD_3O*H* and *H*OD protons (the latter, from the initial content in CD_3OD and humidity) [24−26].
(84) Nyquist, R. A.; Putzig, C. L.; Hasha, D. L. Solvent Effect Correlations for Acetone: IR versus NMR Data for the Carbonyl Group. *Appl. Spectrosc*. **1989**, *43*, 1049−1053.
(85) Handa, M.; Kataoka, M.; Wakaumi, M.; Sasaki, Y. Physical and Donor–Acceptor Properties of 3-Propyl-4-ethylsydnone. *Bull. Chem. Soc. Jpn*. **1997***, 70,* 315−320.
(86) It should be noted that δ_C data for the signals of some NMR solvents have been originally published [24] with quite large uncertainties, *e.g.,* of ±0.06 ppm for $CDCl_3$ and DMSO-d_6.
(87) He, X.; Yan, Z.; Hu, X.; Zuo, Y.; Jiang, C.; Jin, L.; Shang, Y. $FeCl_3$-Catalyzed Cascade Reaction: An Efficient Approach to Functionalized Coumarin Derivatives. *Synth. Commun.* **2014**, *44*, 1507−1514.
(88) Ghadimi, H.; Ghani, S. A.; Amiri, IS. Electrochemistry of Dihydroxybenzene Compounds. Carbon Based Electrodes and Their Uses in Synthesis and Sensors; Elsevier: Amsterdam, 2017, Chapter 3, pp. 47−49.
(89) Breitmaier, E.; Voelter, W. *^{13}C NMR Spectroscopy. Methods and Applications*. 1st ed.; Verlag Chemie GmbH: Weinheim, 1974, p 70.
(90) Pretsch, E.; Clerc, T.; Seibl, J.; Simon, W. *Tabellen zur Strukturaufklärung organischer Verbindungen mit spektroskopischen Methoden*; Springer Verlag: Berlin, 1976, pp. C250−C260.
(91) Pretsch, E.; Clerc, T.; Seibl, J.; Simon, W. *Tabellen zur Strukturaufklärung organischer Verbindungen mit spektroskopischen Methoden*; Springer Verlag: Berlin, 1981, pp. C250−C260.
(92) Breitmaier, E.; Voelter, W. *^{13}C NMR Spectroscopy. Methods and Applications in Organic Chemistry*. 2nd ed.; Verlag Chemie: Weinheim, 1978, p. 69.
(93) Breitmaier, E.; Voelter, W. *^{13}C NMR Spectroscopy. Methods and Applications. 3rd ed.;* VCH Verlagsgesellschaft mbH: Weinheim, 1987, p. 109.
(94) *NMR-Solvents - Online*. https://www.science-and-fun.de/tools/solvents/ (accessed on 21 May 2023).
(95) Pouchert, C. J.; Behnke, J., Eds., *The Aldrich Library of ^{13}C and ^{1}H FT NMR Spectra*; Aldrich Chemical Company, Inc.: Milwaukee, 1993; Vol. 3, pp. 620A−625A.
(96) Jacobsen, N. E. NMR Data Interpretation Explained: Understanding 1D and 2D NMR Spectra of Organic Compounds and Natural Products; John Wiley & Sons Inc.: Hoboken, 2017, p. 130.
(97) Harris, R. K.; Becker, E. D.; de Menezes, S. M. C.; Goodfellow, R.; Granger, P. NMR nomenclature. Nuclear spin properties and conventions for chemical shifts (IUPAC Recommendations 2001). *Pure Appl. Chem.* **2001***, 73,* 1795−1818.
(98) Hoffman, R. (The Hebrew University of Jerusalem, Israel). Personal communications, 2022−2023.
(99) Skorupska, E. A.; Nazarski, R. B.; Ciechańska, M.; Jóźwiak, A.; Kłys, A. Dynamic ^{1}H NMR spectroscopic study of hindered internal rotation in selected *N,N*-dialkyl isonicotinamides: an experimental and DFT analysis. *Tetrahedron* **2013**, *69*, 8147−8154.
(100) Wei, J.; Zhou, X.; Dong, M.; Yang, L.; Zhao, C.; Lu, R.; Bao, G.; Hu, F. Metabolites and novel compounds with anti-microbial or antiaging activities from *Cordyceps fumosorosea*. *AMB Express*, **2022**, *12*, 40.
(101) Manwill, P. K.; Kalsi, M.; Wu, S.; Martinez Rodriguez, E. J.; Cheng, X.; Piermarini, P. M.; Rakotondraibe, H. L. Semi-synthetic cinnamodial analogues: Structural insights into the insecticidal and antifeedant activities of drimane sesquiterpenes against the mosquito *Aedes aegypti*. *PLOS Negl. Trop. Dis.* **2020**, *14*, e0008073.
(102) Grzonka, M.; Davies, A. N. Empirical Investigation on the Reproducibility of ^{13}C NMR Shift Values. *J. Chem. Inf. Comput. Sci.* **1998**, *38*, 1096−1101.
(103) Granger, P.; Bourdonneau, M.; Assémat, O.; Piotto, *M.* NMR Chemical Shift Measurements Revisited: High Precision Measurements. *Concepts Magn. Reson. A* **2007**, *30*, 184−193.
(104) These values are available using the *edlock* command in Bruker's TopSpin software.
(105) Morodo, R.; Riva, R.; van den Akker, N. M. S.; Molin, D. G. M.; Jérôme, C.; Monbaliu, J.-C. M. Accelerating the end-to-end production of cyclic phosphate monomers with modular flow chemistry. *Chem. Sci.* **2022**, *13*, 10699−10706 (Electronic Supplementary Material, p. S65).
(106) *HyperChem^{TM} – Molecular Modeling System, Release 8.0.10 for Windows*; Hypercube, Inc.: Gainesville, FL, 2011.
(107) Kadam, R. U.; Garg, D.; Schwartz, J.; Visini, R.; Sattler, M.; Stocker, A.; Darbre, T.; Reymond, J.-L. CH−π "T-Shape" Interaction with Histidine Explains Binding of Aromatic Galactosides to Pseudomonas aeruginosa Lectin LecA. *ACS Chem. Biol.* **2013**, *8*, 1925−1930 and refs cited therein.

(108) Frisch, M. J.; Trucks, G. W.; Schlegel, H. B.; Scuseria, G. E.; Robb, M. A.; Cheeseman, J. R.; Scalmani, G.; Barone, V.; Petersson, G. A.; Nakatsuji, H.; Li, X.; Caricato, M.; Marenich, A. V.; Bloino, J.; Janesko, B. G.; Gomperts, R.; Mennucci, B.; Hratchian, H. P.; Ortiz, J. V.; Izmaylov, A. F.; Sonnenberg, J. L.; Williams-Young, D.; Ding, F.; Lipparini, F.; Egidi, F.; Goings, J.; Peng, B.; Petrone, A.; Henderson, T.; Ranasinghe, D.; Zakrzewski, V. G.; Gao, J.; Rega, N.; Zheng, G.; Liang, W.; Hada, M.; Ehara, M.; Toyota, K.; Fukuda, R.; Hasegawa, J.; Ishida, M.; Nakajima, T.; Honda, Y.; Kitao, O.; Nakai, H.; Vreven, T.; Throssell, K.; Montgomery, Jr., J. A.; Peralta, J. E.; Ogliaro, F.; Bearpark, M. J.; Heyd, J. J.; Brothers, E. N.; Kudin, K. N.; Staroverov, V. N.; Keith, T. A.; Kobayashi, R.; Normand, J.; Raghavachari, K.; Rendell, A. P.; Burant, J. C.; Iyengar, S. S.; Tomasi, J.; Cossi, M.; Millam, J. M.; Klene, M.; Adamo, C.; Cammi, R.; Ochterski, J. W.; Martin, R. L.; Morokuma, K.; Farkas, O.; Foresman, J. B.; Fox, D. J. *Gaussian 16, Revision C.01;* Gaussian, Inc.: Wallingford, CT, 2019.

(109) *PCMODEL for Windows. Version 8.50.0. Molecular Modeling Software for Windows Operating System, Apple Macintosh OS, Linux and Unix*; Serena Software: Bloomington, IN, September 2003.

(110) Rauhut, G.; Puyear, S.; Wolinski, K.; Pulay, P. Comparison of NMR Shieldings Calculated from Hartree-Fock and Density Functional Wave Functions Using Gauge-Including Atomic Orbitals. *J. Phys. Chem*. **1996**, *100*, 6310−6316.

(111) Tomasi, J.; Mennucci, B.; Cancès, E. The IEF Version of the PCM Solvation Method: An Overview of a New Method Addressed to Study Molecular Solutes at the QM ab Initio Level. *J. Mol. Struct. THEOCHEM* **1999**, *464*, 211−226.

(112) Michalik, E.; Nazarski, R. B. Synthesis, complete NMR assignments, and NOE versus GIAO data assisted ab initio modelling the overall conformations of amide 3,4'-diquinolinyl sulfides in solution. Another approach to analysis of flexible systems. *Tetrahedron*, **2004**, *60,* 9213−9222.

(113) Nazarski, R. B. Summary of DFT calculations coupled with current statistical and/or artificial neural network (ANN) methods to assist experimental NMR data in identifying diastereomeric structures. *Tetrahedron Lett.* **2021**, *71*, 152548 and refs cited therein.

(114) *Chemcraft, Version 1.8 (built 523b)* – A graphical visualization program for quantum chemistry computation.

(115) Makulski, W.; Jackowski, K. ^{1}H, ^{13}C and ^{29}Si magnetic shielding in gaseous and liquid tetramethylsilane. *J. Magn. Reson.* **2020**, *313*, 106716.

(116) Rzepiela, K.; Kaminský, J.; Buczek, A.; Broda, M. A.; Kupka, T. Electron Correlation or Basis Set Quality: How to Obtain Converged and Accurate NMR Shieldings for the Third-Row Elements? *Molecules* **2022**, *27*, 8230.
